# Diversity, duplication, and genomic organization of homeobox genes in Lepidoptera

**DOI:** 10.1101/gr.277118.122

**Published:** 2023-01

**Authors:** Peter O. Mulhair, Liam Crowley, Douglas H. Boyes, Amber Harper, Owen T. Lewis, Darwin Tree of Life Consortium, Peter W.H. Holland

**Affiliations:** 1 Department of Biology, University of Oxford, Oxford OX1 3SZ, United Kingdom;; 2 UK Centre for Ecology and Hydrology, Wallingford OX10 8BB, United Kingdom;; 5 Wellcome Sanger Institute, Wellcome Genome Campus, Hinxton, Cambridgeshire CB10 1SA, UK;; 6 Marine Biological Association of the United Kingdom, Plymouth PL1 2PB, UK;; 7 University of Liverpool, Liverpool L69 3BX, UK;; 8 University of East Anglia, Norwich Research Park, Norwich NR4 7TJ, UK;; 9 Department of Biology, University of Oxford, Oxford OX1 3SZ, UK;; 10 Department of Genetics, University of Cambridge, Cambridge CB2 3EH, UK;; 11 Royal Botanic Gardens, London TW9 3AE, UK;; 12 Royal Botanic Garden Edinburgh, Edinburgh EH3 5LR, UK;; 13 University of Plymouth, Plymouth PL4 8AA, UK;; 14 Institute of Evolutionary Biology, School of Biological Sciences, University of Edinburgh, Edinburgh EH8 9YL, UK;; 15 Natural History Museum, London SW7 5BD, UK;; 16 EMBL-EBI, Wellcome Genome Campus, Hinxton, Cambridgeshire CB10 1SD, UK

## Abstract

Homeobox genes encode transcription factors with essential roles in patterning and cell fate in developing animal embryos. Many homeobox genes, including Hox and NK genes, are arranged in gene clusters, a feature likely related to transcriptional control. Sparse taxon sampling and fragmentary genome assemblies mean that little is known about the dynamics of homeobox gene evolution across Lepidoptera or about how changes in homeobox gene number and organization relate to diversity in this large order of insects. Here we analyze an extensive data set of high-quality genomes to characterize the number and organization of all homeobox genes in 123 species of Lepidoptera from 23 taxonomic families. We find most Lepidoptera have around 100 homeobox loci, including an unusual Hox gene cluster in which the *lab* gene is repositioned and the *ro* gene is next to *pb*. A topologically associating domain spans much of the gene cluster, suggesting deep regulatory conservation of the Hox cluster arrangement in this insect order. Most Lepidoptera have four Shx genes, divergent *zen*-derived loci, but these loci underwent dramatic duplication in several lineages, with some moths having over 165 homeobox loci in the Hox gene cluster; this expansion is associated with local LINE element density. In contrast, the NK gene cluster content is more stable, although there are differences in organization compared with other insects, as well as major rearrangements within butterflies. Our analysis represents the first description of homeobox gene content across the order Lepidoptera, exemplifying the potential of newly generated genome assemblies for understanding genome and gene family evolution.

Lepidoptera (moths and butterflies) are one of the four megadiverse insect orders, with over 150,000 described species. Lepidoptera belong within the Endopterygota, meaning they undergo complete metamorphosis with development proceeding from a motile phytophagous larva to a pupal stage to a reproductive imago (adult). The imaginal stage is easily recognizable and typically follows a characteristic body plan: two pairs of scale-covered membranous wings, six walking legs, filamentous antennae, and a tube-like proboscis. There are, however, variations and exceptions. For example, in some moths, the females are flightless with reduced wings; in many butterflies, four legs rather than six are used for walking; antennal morphology varies with clubbed, lamellate, or plumose structure; in several moth species, the larvae are fully aquatic; and in the family Micropterygidae, the adults have biting rather than sucking mouthparts. Many variations and adaptations are hypothesized to have been driven by coevolution with plants, driving novelties in egg laying behavior, larval phenotype, and feeding strategies in both larvae and adults (Wiens et al. 2015; Mitter et al. 2017; Kawahara et al. 2019).

Associating evolutionary change in form or behavior to changes in underlying loci is not straightforward, but insights can come from correlations between patterns in molecular evolution and changes in phenotype. Homeobox genes are candidates for loci in which molecular change may cause or facilitate evolutionary change to the form and structure of animals, because most homeobox genes play regulatory roles in development. For example, the Hox genes, a subset of homeobox genes, encode transcription factors that control spatial identity along the anteroposterior axis in embryonic development, and their number differs between animal lineages. There was an increase in Hox gene number on the stem lineage of bilaterian animals, when a head-to-tail axis evolved to dominate the body plan (Finnerty and Martindale 1998; Holland 2015; Nong et al. 2020); there was also an increase in the early evolution of vertebrates, traceable to genome duplication (Soshnikova et al. 2013; Aase-Remedios and Ferrier 2021). Hox genes are usually arranged in gene clusters, but these clusters have been secondarily broken or dispersed in some evolutionary lineages concomitant with changes to developmental pathways (Ferrier and Holland 2002); conversely, clusters have been further compacted in vertebrates in association with additional gene regulatory controls and the emergence of fins and limbs (Duboule 2007). We wished to address if changes to the homeobox complement in Lepidoptera were associated with phenotypic change.

In Lepidoptera, lack of high-contiguity, chromosomal-scale genome assemblies have hampered studies into the structure and evolution of the Hox gene cluster, so the extent of gene cluster compaction, cluster integrity, and precise gene order remains unclear. One discovery was the presence of at least 11 divergent homeobox loci within the Hox gene cluster of the silkworm, *Bombyx mori* (Chai et al. 2008), all located between the *zen* and *pb* Hox genes. This presence of unusual “special homeobox” (Shx) genes within the Hox gene cluster was later confirmed in several other Lepidoptera, most of which were found to possess four Shx genes, *ShxA*, *ShxB*, *ShxC*, and *ShxD*, derived by tandem duplication and divergence from *zen* (Ferguson et al. 2014). These studies also highlighted *B. mori* as an aberrant outlier to the usual pattern, with the larger number of Shx genes reflecting further tandem duplication of *ShxD*. *Triodia sylvina* (orange swift moth, family Hepialidae) was also noted as unusual, as it seemed to lack Shx genes altogether, although tentative evidence for *zen* duplication was found (Ferguson et al. 2014). We wished to refine when Shx genes arose and to also test if Shx expansion in *Bombyx* is unique.

Although the roles of Shx genes are not yet fully understood, studies in *Pararge aegeria* (speckled wood butterfly) have shown expression in the extraembryonic serosa and suggested functions in extraembryonic membrane patterning (Ferguson et al. 2014). It should also be noted that relatively few species were compared in these initial surveys owing to the lack of genomic data; hence, patterns of Shx gene evolution were poorly resolved. Outside of the Hox gene cluster, even less is known about the evolution of homeobox genes across Lepidoptera. For example, the NK genes are members of the ANTP class, like Hox genes, and are arranged in a compact gene cluster in Diptera and Coleoptera (Jagla et al. 2001; Garcia-Fernàndez 2005; Butts et al. 2008); these genes are implicated in mesoderm development, but their evolution has not been analyzed comprehensively in Lepidoptera (Ranz et al. 2022). The same can be said for the many dispersed homeobox genes that are not arranged in gene clusters and are implicated in a wide diversity of developmental roles (Ferrier 2016). We aimed to assess the extent of homeobox gene clustering in Lepidoptera, beyond the Hox cluster.

Until recently, analysis of the copy number, organization, and molecular evolution of homeobox genes across a whole insect order has not been feasible owing to limited sampling of species and, for the study of clustered homeobox genes, the highly fragmented nature of many genome assemblies. Dense sampling of lepidopteran species in the Darwin Tree of Life Project (The Darwin Tree of Life Project Consortium 2022) has generated chromosome-level genome assemblies across a wide phylogenetic coverage. Analyzing these data, we present an order-wide description of the homeobox gene content in Lepidoptera. Using chromosome-level genome assemblies for 123 lepidopteran genomes from 23 taxonomic families, we identified all homeobox genes from their characteristic homeodomain, determined their genomic organization into gene clusters, and traced their patterns and pathways of duplication and loss.

## Results

### Classification of all Lepidoptera homeobox genes

**Figure 1.**
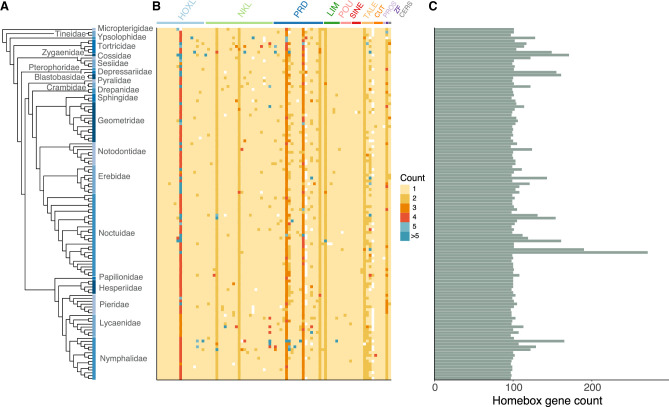
Numbers of homeobox sequences across Lepidoptera. (*A*) Species tree of Lepidoptera analyzed constructed using BUSCO gene set. Colored boxes spanning tips of the tree represent distinct Lepidoptera families with the family names shown. Species in the tree are listed in the same order as in Supplemental Figure S1. (*B*) Heatmap showing numbers of homeobox loci in each gene class and subclass (from *left* to *right*: *lab*, *Abd-B*, *abd-A*, *Ubx*, *Antp*, *ftz*, *Scr*, *Dfd*, *zen*, *Shx*, *pb*, *ind*, *cad*, *exex, eve, unpg, btn, Tlx, Msx, NK4, NK3, Lbx, NK1, Hmx, Emx, Hhex, NK7, NK6, Nedx, Dlx, En, NK2.1, Msxlx, Hlx, NK2.2, Barhl, Bari, Bsx, Dbx, Abox, Noto, ro, Uncx, Gsc, Pitx, Otp, Rx, Hbn, Repo, Prrx, Shox, Arx, Pax4/6, Phox, Prop, Vsx, CG11294, Pax3/7, Drgx, Otx, Lhx6/8, Lmx, Lhx2/9, Lhx3/4, Lhx1/5, Isl, Pou2, Pou3, Pou4, Pou6, Six3/6, Six1/2, Six4/5, Meis, Irx, Mkx, Pbx, Tgif, Onecut, Cux, Cmp, Prox, Zfhx, Cers*). (*C*) Total counts of homeobox loci in each genome.

We identified all homeobox gene loci in the genomes of 123 lepidopteran species, including 87 moths and 36 butterfly species (Supplemental Table S1; http://doi.org/10.5281/zenodo.7274111). To place our analyses in an evolutionary context, we also constructed a phylogenetic tree of the species analyzed using 2262 BUSCO genes (see Methods; Fig. 1A; Supplemental Fig. S1). Homeobox sequences were then classified using the characteristic homeodomain and a combination of reciprocal best BLAST and molecular phylogenetic analysis: This “total” collection of homeobox loci could include functional genes, partial genes, and pseudogenes.

We find that the catalog of homeobox loci is relatively stable across Lepidoptera (Fig. 1B,C), with most species possessing around 100 homeobox sequences. However, certain lineages and species showed marked increases in homeobox counts, resulting mainly from duplications within individual homeobox gene classes. The main contributors to these increases are large expansions within the Hox gene cluster in some clades or smaller-scale duplications of PRD class genes. Homeobox gene loss has also occurred. For example, the *HHEX (Hhex)* gene of the ANTP class is absent from the genomes of all three *Pieris* species sequenced, consistent with a loss in this clade (Fig. 1B). We deduce that the *ShxD* gene was lost in the genus *Melitaea*, as it is absent in both *Melitaea cinxia* (Glanville fritillary butterfly), consistent with an earlier report (Ferguson et al. 2014), and *Melitaea athalia* (heath fritillary butterfly). Similarly, we do not find the *ShxD* gene in any of the eight Lycaenidae species in our data set (*Lycaena phlaeas, Celastrina argiolus*, *Glaucopsyche alexis*, *Plebejus argus*, *Cyaniris semiargus*, *Aricia agestis*, *Lysandra bellargus*, and *Lysandra coridon*), implying that this gene was also lost early in the evolution of the family Lycaenidae. Some homeobox genes, such as *Mkx* (orthologous to Dmel\*CG11617*) of the TALE class, were lost many times independently across Lepidoptera (Fig. 1B).

Using a representative set of seven species, we examined expression levels for each homeobox gene using female whole-body RNA-seq (Supplemental Fig. S2). We find clear evidence for the expression of Hox genes and Shx genes, with particularly strong expression of *ShxC*; consistent expression of homeobox genes in the SINE, TALE, CUT, PROS, ZF and CERS classes; and variable expression of PRD class and NK homeobox genes.

### Rearrangement of the Hox gene cluster

Within insects, the Hox gene cluster generally comprises 10 homeobox genes arranged in a specific order reflecting their evolutionary origin by tandem gene duplication: *lab*, *pb*, *zen*, *Dfd*, *Scr*, *Antp*, *ftz*, *Ubx*, *abd-A*, and *Abd-B*. The cluster may be split, as in many *Drosophila* species (Duboule 2007; Negre and Ruiz 2007), individual genes may be inverted, and the *zen* gene may be duplicated (e.g., *zen*, *zen2*, and *bcd* in *Drosophila melanogaster*), but radical gene order changes are rare, documented only within individual species or close relatives (Negre et al. 2005). A difficulty in studying gene order is that intergenic distances may be large, and many genome assemblies do not provide long-range linkage information. Using chromosome-level gene assemblies (Supplemental Table S1), we have determined the structure of the Hox gene cluster in 123 Lepidoptera genomes, providing the first comprehensive description of the cluster evolution across this order.

**Figure 2.**
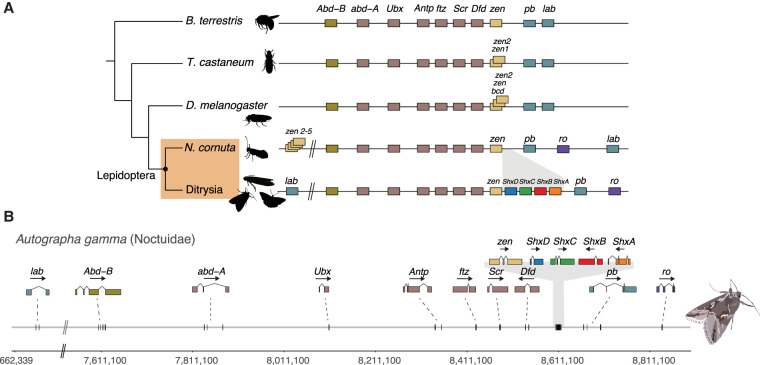
Hox gene cluster evolution across Insecta. (*A*) Comparison of the general structure of the Hox gene cluster between representative species for Hymenoptera (*Bombus terrestris*), Coleoptera (*Tribolium castaneum*), Diptera (*Drosophila melanogaster*), and Lepidoptera. Lepidoptera are shaded in an orange box and split between non-Ditrysia species (*Neomicropteryx cornuta*) and Ditrysia (represented by 122 species in our data set). Lepidoptera-specific Shx genes are colored orange (*ShxA*), red (*ShxB*), green (*ShxC*), and blue (*ShxD*) in this figure and throughout the paper. (*B*) Genomic location of Hox genes in *A. gamma* with corresponding exon structures and genomic distances annotated *below*. Silhouette images of *B. terrestris*, *T. castaneum*, and *D. melanogaster* were taken from PhyloPic (phylopic.org).

We found all Hox genes on a single scaffold for 115/123 genomes (Supplemental Fig. S3). In all Lepidoptera we analyzed, we found the canonical *lab*, *pb*, *Dfd*, *Scr*, *Antp*, *Ubx*, *abd-A*, and *Abd-B* homeotic genes, plus the divergent Hox-derived genes *zen* and *ftz*, along with gene order, orientation, and intergenic distances. In most Lepidoptera, excepting some “basal” lineages, we also found four distinct Shx genes (*ShxA* to *ShxD*) between *zen* and *pb* (Figs. 1, 2; Supplemental Fig. S3), as previously noted for a smaller sample of species (Ferguson et al. 2014). The structure of the Hox cluster for *Autographa gamma* (silver Y moth) (Boyes et al. 2022c) shown in Figure 2B reflects the general structure found in most lepidopteran species.

When compared with other insect orders, two rearrangements are apparent. First, we consistently find a non-Hox homeobox gene, rough (*ro*), in close association with the gene cluster. Across almost all Lepidoptera species, the *ro* gene is adjacent to *pb*, in the genomic location where *lab* or its ortholog is found in most species (shown for *A. gamma* in Fig. 2). Second, the *lab* gene has been translocated to a distant genomic location beyond *Abd-B*. This dissociation of *lab* is consistent with a split between *lab* and other Hox genes previously reported in *B. mori*, although the position of *lab* was unresolved in this earlier work (Yasukochi et al. 2004; Chai et al. 2008). In *A. gamma*, the *lab* gene is ∼7 Mb from *Abd-B*, whereas the main part of the Hox cluster spans 1.22 Mb from *Abd-B* to *ro*. We find the Hox gene cluster (excluding *lab*) in Lepidoptera ranges from 1 Mb in *Papilio machaon* (swallowtail butterfly) to 6.8 Mb in *Euproctis similis* (yellow-tail moth). A further inversion of the *ro* gene occurred within the *Pieris* clade, resulting in relocation of *ro* to between *lab* and *Abd-B* (Supplemental Fig. S3).

### An evolutionarily conserved topologically associating domain around the Hox cluster

**Figure 3.**
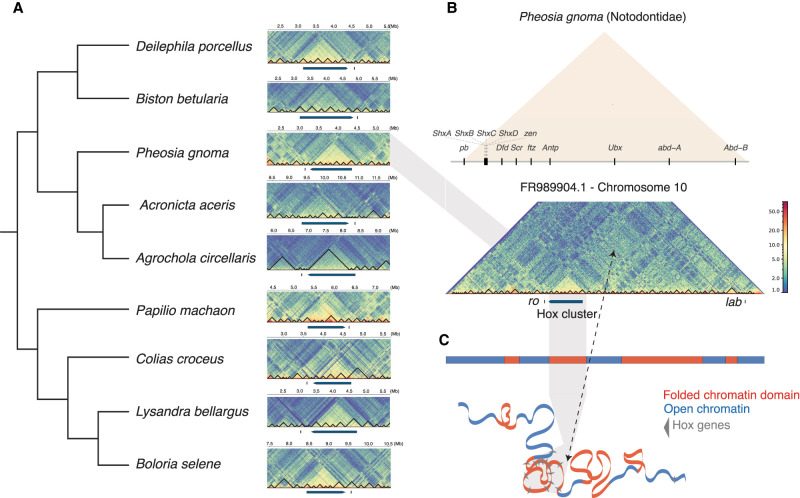
Evidence for a conserved topologically associated domain (TAD) spanning the Hox gene cluster across Lepidoptera. (*A*) Species tree of nine representative lepidopteran species on *left*; Hi-C matrix showing 1 Mb either side of the Hox gene cluster (excluding *lab*). The location of the Hox gene cluster from (*Abd-B* to *pb*) is annotated by a blue bar, along with its orientation. The position of *ro* is annotated with a short vertical black dash. The intensity of chromatin compaction is represented by a blue (low) to red (high) color gradient. Across the core Hox cluster (*pb* to *Abd-B*) in each species a TAD is represented by a region of strong contact, by the more-yellow-shaded regions. Black lines represent TADs or sub-TADs predicted by HiCExplorer. (*B*, *top*) The arrangement of the Hox gene cluster (excluding *ro* and *lab*) surrounded by a TAD (orange) in *Pheosia gnoma* (lesser swallow prominent). (*Bottom*) The Hi-C matrix of Chromosome 10 showing the location of the Hox cluster, represented by a blue bar, along with *ro* and *lab*, represented by short vertical black dashes. (*C*) Schematic showing tologically folded domains in Chromosome 10 (red) interspersed by chromosome regions with less consistent topology (blue) based on the above Hi-C matrix. Shaded gray region shows the location of the condensed TAD containing the Hox cluster.

To assess whether the rearrangements in gene order could be associated with changes in regulation of Hox genes, we used Hi-C data to annotate topologically associating domains (TADs) across the genome. These data can reveal the 3D organization of the chromatin and, at least in some cases, highlight regions of the genome under common regulatory constraints (Schoenfelder and Fraser 2019; Szabo et al. 2019). Given that the purpose of the Hi-C sequencing of these species was to assist in genome assembly (Lawniczak et al. 2022), the depth of Hi-C is lower than in some other studies (Liao et al. 2021), ranging from around 35 million to 52 million paired-end reads (Supplemental Table S2). Nonetheless, we found this sequencing depth sufficient for the analysis, revealing TADs in lepidopteran genomes, which were visualized at 5-kb resolution using HiCExplorer (Ramírez et al. 2018). To our knowledge, this is the first such analysis for Lepidoptera genomes and is one of the few assessments of chromatin accessibility around invertebrate Hox gene clusters (Acemel et al. 2017). In a sample of nine species representing diverse families across Lepidoptera (Fig. 3A), we observe strong evidence of an evolutionarily conserved, prominent TAD covering most of the Hox gene cluster from *pb* to *Abd-B* (Fig. 3A,B). This TAD was also observed in species with a large increase in copy number within the Hox cluster (see section “Independent tandem duplication of Shx genes” below) (Supplemental Fig. S4A). In all species analyzed, *lab* and *ro* are located outside the distinct TAD (Fig. 3A). Assessing the wider chromosomal organization in *Pheosia gnoma* (lesser swallow prominent), it is clear that there is a high degree of contact within the Hox-containing TAD relative to the rest of the chromosome (Fig. 3B). Although genome-wide conservation of TADs between species has been questioned (Eres and Gilad 2021), we argue that the strong and consistent signal for physical contacts across the Hox cluster in diverse moths and butterflies is evidence for a conserved TAD around a cluster of developmentally important genes. We make no assessment of possible conservation of other TADs in lepidopteran genomes. Outside the Hox gene cluster, the general structure of TADs across this chromosome appears similar to the pattern observed in *Drosophila*, with TADs representing condensed internal interactions and larger compartments showing long-range interactions between these domains (Fig. 3B,C; Sexton et al. 2012; Ulianov et al. 2016; Szabo et al. 2018; Liao et al. 2021).

### Origin, duplication, and loss of Shx genes

**Figure 4.**
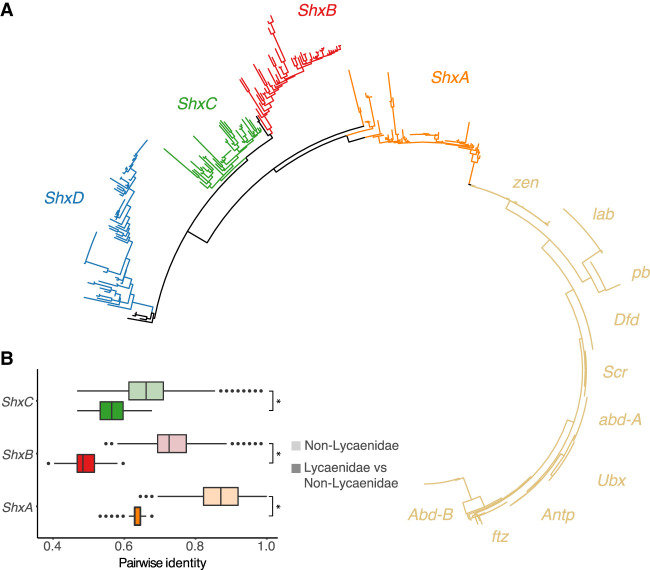
Sequence evolution of Hox genes across Lepidoptera. (*A*) Phylogenetic tree of Hox and Hox-derived homeodomains across 46 Lepidoptera species. Shx gene clades are colored orange, red, green, and blue; canonical Hox genes are colored yellow. The names of the Hox genes are placed alongside their clade in the tree. (*B*) Shx genes show elevated sequence evolution following loss of *ShxD* in Lycaenidae. Results of pairwise identity of Shx genes between Lycaenidae species and non-Lycaenidae species. For each gene (*ShxA–C*), pairwise identity between Lycaenidae and all other Lepidoptera species with normal Shx gene count (darker shade boxplot) is compared with pairwise identity between all Lepidoptera species with normal Shx gene count (lighter shade boxplot). Each pair of boxplots (light shade and dark shade) are colored according to the color code for each of the Shx genes. Wilcoxon rank-sum test was performed between pairwise identity for Lycaenidae and non-Lycaenidae species: (*) *P*-value < 0.05.

As in most Lepidoptera, four distinct Shx genes were identified between *zen* and *pb* in the Hox gene cluster of *A. gamma* (Fig. 2; Supplemental Fig. S3). Phylogenetic analysis supports the derivation of Shx genes by tandem duplication and sequence divergence from *zen* (see Fig. 4A; Ferguson et al. 2014). We also find rapid sequence divergence within the homeodomain of these genes following duplication from *zen*, as previously described (Fig. 4A; Ferguson et al. 2014). Shx genes are found in representatives of the Erebidae, Nymphalidae, Sphingidae, Noctuidae, Lycaenidae, Pieridae, Papilionidae, Notodontidae, Drepanidae, Hesperiidae, Tortricidae, Geometridae, Sesiidae, Blastobasidae, Depressariidae, Crambidae, Pterophoridae, Pyralidae, Tineidae, Ypsolophidae, Cossidae, and Zygaenidae families, but we do not identify these genes in Micropterigidae (Fig. 2A; Supplemental Fig. S3). Instead, extra copies of *zen* (four in addition to the original *zen*) were found in a species from the family Micropterigidae (*Neomicropteryx cornuta*). These loci, which are located outside the Hox cluster beyond the location of *Abd-B*, group outside of the Shx genes in a molecular phylogenetic analysis: two with the *zen* clade and three closer to the *lab* clade (Fig. 4A). We suggest all are derived from *zen*. They display higher rates of substitution in the homeodomain compared with the other *zen* genes analyzed, which may underlie erroneous placement of some genes closer to *lab*.

The *ShxD* gene was lost several times across the lepidopteran phylogeny. One loss event is shared by the Lycaenidae species (the “blue” butterflies), suggesting gene loss along the ancestral branch of this diverse family. Loss of *ShxD* in these species is associated with longer branch lengths in the remaining Shx genes (*ShxA*-*C*) in a phylogenetic analysis (Fig. 4A). The significantly increased rate of substitution in the homeodomain of the three remaining Shx genes (*ShxA*, *ShxB*, and *ShxC*) following the loss of the *ShxD* gene was confirmed by assessing pairwise sequence identity between Lycaenidae species and non-Lycaenidae species (Fig. 4B).

### Independent tandem duplication of Shx genes

As noted above, the number of Shx genes in ditryisian lepidopterans is usually four, or three in those taxa that have lost *ShxD*. However, there are some notable examples of Shx gene duplication. In earlier work using limited sampling and fragmentary genome assemblies, the large number of Shx loci in *B. mori* was considered an exception to the normal pattern (Chai et al. 2008; Ferguson et al. 2014). The expanded sampling generated by the Darwin Tree of Life Project reveals a more complex pattern of evolution. Although presence of four Shx genes is still the norm for Lepidoptera, we find multiple independent examples of dramatic Shx gene number expansion (Fig. 1B; Supplemental Fig. S3). In 18 species of moth (*Zeuzera pyrina*, *Blastobasis lacticolella*, *Blastobasis adustella*, *Parapoynx stratiotata*, *Idaea aversata*, *Phalera bucephala*, *E. similis*, *Schrankia costaestrigalis*, *Spilarctia lutea*, *Spilosoma lubricipeda*, *Eilema depressum*, *Eilema sororculum*, *Mythimna ferrago*, *Mythimna impura*, *Noctua pronuba*, *Noctua janthe*, *Noctua fimbriata*, and *Apamea monoglypha*) and one butterfly species (*Aporia crataegi*), a large number of homeobox loci was found between *zen* and *pb*, each representing extensive tandem duplication of Shx genes (Supplemental Figs. S2, S5). In these species, the copy number ranges from nine copies of Shx genes in *N. pronuba* to 51 Shx copies in *M. impura*, up to 165 Shx loci in *A. monoglypha*, the largest number observed. These species have a mean of 32 copies of *zen*/Shx and a median of 20 copies. The rate of sequence divergence of the Shx genes following tandem duplication varies between species, with tandemly duplicated copies in three species showing significantly lower pairwise identity (larger sequence change compared with the distribution of pairwise identity in nonduplicated orthologs), duplicated copies from three species showing higher pairwise identity (possibly reflecting recent duplication), and four species showing no significant difference.

This shows that the Shx expansion phenomenon is more widespread across Lepidoptera than previously recognized. In some cases, we observe tandem duplication of Shx genes in closely related species, for example, two *Blastobasis* species, three *Noctua* species, and two *Mythimna* species, suggesting that these events occurred in the common ancestor of each of these lineages or that these lineages are prone to Shx duplication. In total, we detect at least 11 cases of independent expansion of the Shx genes in addition to the previously recognized *B. mori* expansion. We rarely see clearly intermediate cases: We detect either a conservative pattern of three to six Shx genes or a dramatically expanded set of Shx genes.

**Figure 5.**
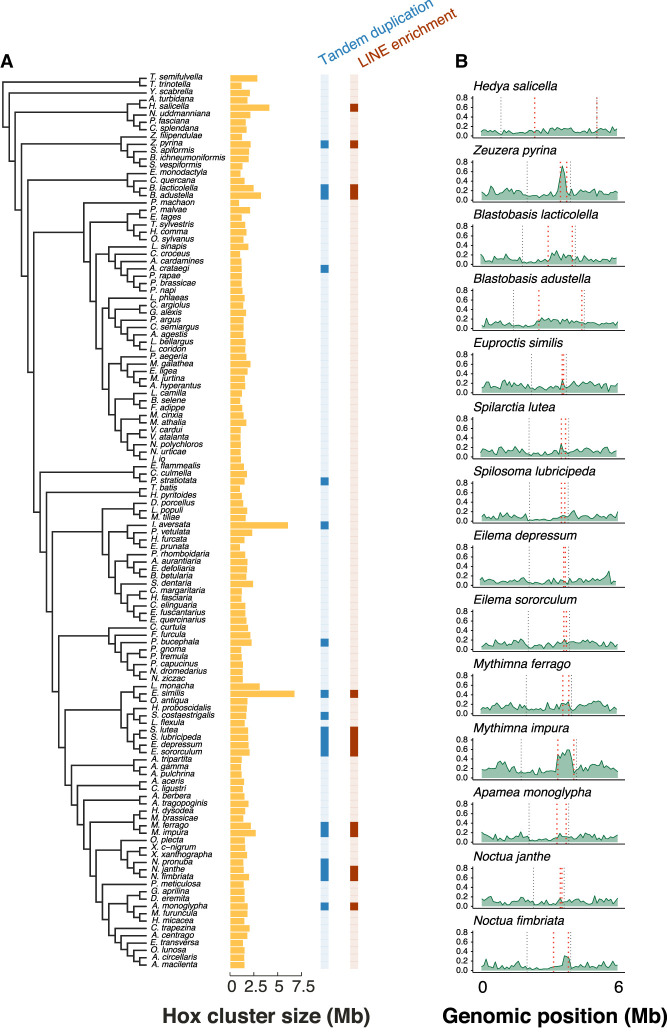
Association between increased LINE density and extensive tandem duplication of Shx genes. (*A*) *Left* shows the species tree of 122 Lepidoptera species. Bar chart in yellow corresponds to the length of the Hox cluster (excluding labial) for each species in the tree measured in Megabases. The column in blue indicates those species with large tandem duplications of Shx genes in the Hox cluster (dark blue) or those with a “normal” number of Shx genes (light blue). The column in red indicates species with significantly enriched density of LINE elements (dark red) within the region containing the Shx genes. (*B*) LINE density plot across the Hox cluster plus 3 Mb either side; this is shown for 14 species with enriched LINE density in the region containing Shx genes. The *outer* black dashed lines represent the edges of the Hox cluster (*Abd-B* to *ro*), and the *inner* red dashed lines represent the edges of the Shx genes (*ShxD* to *ShxA*).

We investigated whether retrotransposon activity may have impacted the copy number variation observed. Retrotransposons, particularly LINE elements, can facilitate nonallelic homologous recombination, resulting in segmental duplications and gene cluster expansions (Startek et al. 2015; Janoušek et al. 2016; Thybert et al. 2018). Repeat content across the whole genomes of 66 representative species was estimated using a combination of RepeatModeler and RepeatMasker pipelines (see Methods; Supplemental Fig. S6). To test the relation between transposon activity and the Hox gene cluster, transposable element (TE) density was annotated in windows of 5000 bases within the Hox cluster (*lab* was excluded from this analysis owing to its distant position). The density of the major classes of TEs (LINEs, SINEs, LTR, and DNA) was compared between the region containing the Shx genes and the remaining Hox cluster. A significantly increased density of the LINE elements was observed within the Shx gene region relative to the rest of the Hox gene cluster in 14 of 19 species with large tandem duplications (Wilcoxon rank-sum test; *P* < 0.05, Bonferroni correction) (Fig. 5A). These 14 species were *Z. pyrina*, *B. lacticolella*, *B. adustella*, *E. similis*, *S. lutea*, *S. lubricipeda*, *E. depressum*, *E. sororculum*, *M. ferrago*, *M. impura*, *N. janthe*, *N. fimbriata*, and *A. monoglypha* (Fig. 5B). Further examining the correlation between Shx expansion and LINE proliferation in the species *Z. pyrina*, which has 25 copies of *ShxA* (Supplemental Figs. S3, S7A), we see that there is clear evidence for tandem duplication of specific LINE elements (LINE/CR1), which are all in the same orientation and evenly interspersed between the *ShxA* copies (Supplemental Fig. S7B). The five species with large tandem duplications but no LINE enrichment were *N. pronuba*, *P. stratiotata*, *Idaea inversata*, *P. bucephala*, and *A. crataegi*. For several species, there is clear evidence that repeat elements were tandemly duplicated along with Shx loci. For example, *P. stratiotata*, with 16 *ShxD* copies, has a repeated array of Low_complexity, Simple_repeat, and LINE/L2 elements between each ShxD. Quite different patterns are seen in *P. bucephala* and *Z. pyrina*. There is no association, beyond the presence of LINEs, between repeat type, repeat number, and which Shx gene is duplicated.

### Other homeobox gene clusters

The Hox genes are the best-studied clustered homeobox genes, but other examples also occur. A cluster of three neuronally expressed homeobox genes from the PRD class—Homeobrain (*hbn*), Retinal Homeobox (*Rx*), and Orthopedia (*otp*)—has been conserved in most animal lineages since the cnidarian–bilaterian ancestor (Mazza et al. 2010). The gene cluster has also been found in *Drosophila* and representatives of Hymenoptera and Coleoptera, with a conserved gene order and comparable intergenic distances (Walldorf et al. 2000; Mazza et al. 2010). Across the lepidopteran species in this study, we also find that the cluster is conserved with the same gene order (Supplemental Fig. S8). Genomic distances between genes are larger in lepidopteran species than in other insects studied to date, with an average overall cluster length of 348 kb. Although gene order is conserved, transcriptional orientation varies between species.

**Figure 6.**
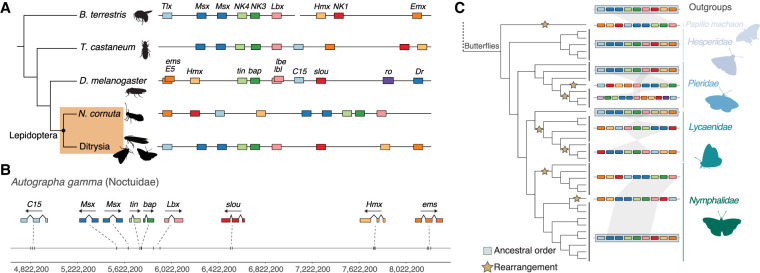
NK gene cluster evolution across Insecta. (*A*) Comparison of the general structure of the NK gene cluster between representative species for Hymenoptera (*B. terrestris*), Coleoptera (*T. castaneum*), Diptera (*D. melanogaster*), and Lepidoptera. Lepidoptera are shaded in an orange box and split between non-Ditrysia species (*N. cornuta*) and Ditrysia (represented by 122 species in our data set). (*B*) Genomic location of NK genes in *A. gamma* with corresponding exon structures and genomic distances annotated *below*. Silhouette images of *B. terrestris*, *T. castaneum*, and *D. melanogaster* were taken from PhyloPic (phylopic.org). (*C*) *Left* shows the species topology for the 36 butterflies in the data set, along with an outgroup representative. Rearrangements in the NK cluster are annotated on the branches of the tree where they were estimated to have occurred (represented by yellow stars). Black lines spanning tips on the tree group species, which show the same structure and order in the NK gene cluster. The NK gene cluster is represented by colored boxes, in the “canonical” order of *Tlx* (*C15*), *Msx* (*Dr*), *NK4* (*tin*), *NK3* (*bap*), *Lbx* (*lbe*), *NK1* (*slou*), *Hmx* (*NK5*), and *Emx* (*ems*). Species with the NK genes in this order are shadowed by a blue box. Synteny between the closely linked genes of both *Msx* (*Dr*) genes, *NK4* (*tin*), *NK3* (*bap*), and *Lbx* (*lbe*) is represented by shaded blocks to show changes in the order and structure of the NK cluster.

In *Drosophila*, several “NK” genes form a compact homeobox gene cluster comprising *tin* (also known as *NK4*), *bap* (also known as *NK3*), two “*Lbx*” genes (*lbl*, *lbe*), *C15* (*Tlx*), and *slou* (also known as *NK1*) (Jagla et al. 2001; Luke et al. 2003; Garcia-Fernàndez 2005). Other NK-related genes are found more distantly and may have been translocated away, including *Dr* (*Msx)*, *ems* (*Emx*), and *Hmx* (*NK5*). Other groupings of NK genes are found in other animal genomes (Jagla et al. 2001; Luke et al. 2003; Garcia-Fernàndez 2005). In contrast to Hox gene clusters, we find the NK gene cluster has undergone extensive gene order changes during insect evolution (Fig. 6A). Across all insect orders, we find tight linkage between *tin*, *bap*, and *Lbx*; we also find *Dr* is closely linked in several orders, but not in Diptera represented by *Drosophila*. Outside these genes, there is considerable variation between orders.

The organization of the NK gene cluster in *A. gamma* (silver Y moth) is typical for Lepidoptera (Fig. 6B). We find a “core” of five homeobox genes (two *Msx*, *tin*, *bap*, and *Lbx*) spanning ∼370 kb, plus linkage to *C15* on one side and *slou*, *Hmx*, and *ems* on the other (Fig. 6B). The arrangement of these genes is generally conserved across most Lepidoptera species (Fig. 6; Supplemental Fig. S9). However, rearrangements within the cluster are observed in some butterfly lineages. For example, in the three *Pieris* species, the order of the *tin*/bap/*Lbx*/*Dr* core cluster is inverted in all species, *C15* is found on a separate chromosome, and the *Abox* and *Bari* homeobox genes are located close to each end of the cluster (Fig. 6C; Supplemental Fig. S9). Rearrangements are also found in both Lycaenidae and Nymphalidae, with different gene orders suggesting independent rearrangements (Fig. 6C). We infer that a series of translocation and inversion events has occurred independently. In lineages such as the *Pieris* butterflies, these changes in the structure of the NK gene cluster reflect general trends of genome remodeling (Hill et al. 2019). The changes within the NK cluster within butterflies represent at least seven likely rearrangement events, contrasting to the general stability in gene order observed in the Hox cluster. Rearrangements were also found outside the butterflies, with independent changes seen in *Ypsolopha scabrella*, *Emmelina monodactyla*, *Carcina quercana*, *Clostera curtula*, *Laspeyria flexula*, *Abrostola tripartite*, and *N. cornuta*. These NK cluster rearrangements in moths include translocation of one, two, or three of the *slou*/*Hmx*/*ems* genes to the opposite end of the cluster, as well as relocation of *C15* to the opposite end of the cluster in five of the seven species (Supplemental Fig. S9). In contrast to the Hox gene cluster, the Hi-C contact data do not provide evidence for a strong TAD spanning the NK homeobox gene cluster (Supplemental Fig. S4B).

## Discussion

### Overall stability of homeobox gene numbers

Although the expression, function, and evolution of homeobox genes has been extensively studied in insects, few studies have made comparisons across an entire insect order. In addition, most studies have focused on Hox genes, with less attention paid to the many other types of homeobox genes or to genomic organization. To a large degree, this is a consequence of the limited number of high-quality chromosomal-level genome assemblies available until very recently. With advances in DNA sequencing technology, coupled with scaffolding using Hi-C, this limitation is being overcome (The Darwin Tree of Life Project Consortium 2022). To better understand homeobox gene evolution in Lepidoptera, we annotated genes from all homeobox classes in 123 well-assembled Lepidoptera genomes.

We found general stability in homeobox gene numbers across the order, with most species having approximately 100 homeobox loci from all classes. This overall consistency in homeobox gene content may relate to overall body plan stability across Lepidoptera. There are some notable variations in gene content between species and families; most of these concern the Hox genes, including the Shx genes, discussed below. Otherwise, we see a degree of consistency, in gene number if not in gene organization. Leaving Hox genes aside, most homeobox genes are dispersed in these genomes, and linkages are not conserved. The NK homeobox genes and the Homeobrain, Retinal Homeobox, and Orthopedia genes from the PRD class are an exception, with both sets of genes having a conserved cluster arrangement in Lepidoptera. The NK cluster usually contains nine genes and spans 2.4 Mb to 10 Mb. In these genes, we find tight clustering across insects of *Msx* (*Dr*), *NK4* (*tin*), *NK3* (*bap*), and *Lbx* (*lbe*), suggestive of a functional constraint or common regulation, whereas the remaining genes *Tlx* (*C15*), *NK1* (*slou*), *Hmx* (*NK5*), and *Emx* (*ems*) have more variation in their gene order. The Homeobrain, Retinal Homeobox, and Orthopedia cluster is a compact cluster, with an average length of ∼300 kb. The genes are highly conserved in order but vary in gene orientation across Lepidoptera.

### The unusual lepidopteran Hox gene cluster

Hox genes are arranged in genomic clusters as a result of tandem gene duplication, followed by selective pressure that has kept Hox genes together as neighbors for hundreds of millions of years. The nature of the selective pressure is not fully understood but may in part be related to long-range regulatory elements important for spatial colinearity of gene expression (McGinnis and Krumlauf 1992; Duboule and Morata 1994; Lemons and McGinnis 2006). Some changes to the structure of the Hox gene cluster have been found in insects (Lewis 1978; Duncan 1987; Ferrier and Akam 1996; Powers et al. 2000; Brown et al. 2002; Negre and Ruiz 2007), and some larger rearrangements were observed in noninsect arthropods (Cook et al. 2001; Grbić et al. 2011; Chipman et al. 2014; Pace et al. 2016; Leite et al. 2018), but we have a fragmentary picture of insect Hox cluster evolution thus far. Indeed, within Lepidoptera, the complete structure of a Hox gene cluster has not been reported; even in the pioneering studies on *B. mori* Hox genes, the precise location of the labial gene could not be resolved (Yasukochi et al. 2004; Chai et al. 2008). With the availability of chromosomal-level genome assemblies, this picture is changing. This study attempts to characterize Hox gene cluster evolution in an insect order on a large scale. Among the findings were (1) determining that the labial gene is located at a distant position beyond *Abd-B*, likely relocated by an inversion event, and (2) the finding that the non-Hox gene *ro* is very closely linked to *pb*, in the position where labial is found in other insects. These two features are seen in all the ditrysian Lepidoptera we analyzed, with an intermediate situation found in *N. cornuta*, a member of the Micropterygidae. This basal moth has a gene order of *pb*, *ro*, and *lab*, suggesting that movement of *ro* into the Hox gene cluster occurred in an ancestor of extant Lepidoptera, whereas the inversion that moved the *lab* gene was a later event. However, even in *N. cornuta*, the *lab* gene is 3.8 Mb from the end of the cluster, suggesting that it had already “escaped” from common control in the earliest Lepidoptera.

What could have allowed these rearrangements in Lepidoptera? One hypothesis is that all functional reasons for maintaining Hox gene clustering have been lost in Lepidoptera, and random rearrangements have been permitted in evolution. An alternative hypothesis is that it is just the *lab* gene that has been permitted to “escape,” perhaps owing to loss of common regulatory control. Our analysis of topologically associated domains (TADs), and comparison to the NK gene cluster, suggests the second hypothesis is most likely. We found a pattern of physical association of chromatin containing the Hox gene cluster, but only from *pb* to *Abd-B*. We find that *lab* and *ro* are located outside of this TAD across all species sampled. This suggests that it is the *lab* gene specifically that has escaped from any common regulation or control; there is evidence that the remaining Hox genes maintain physical association in three dimensions and are thus under conserved regulation (Krefting et al. 2018). Similarly, although the *ro* gene has moved to be adjacent to the rest of the Hox cluster, it has not been encompassed within the same TAD. Consistent with this conclusion, the *ro* gene has moved secondarily to the *Abd-B* end of the Hox cluster in four closely related Pieridae species (*A. crataegi*, *Pieris rapae*, *Pieris brassicae*, and *Pieris napi*) (Supplemental Fig. S3).

### Moths take the record for the most Hox loci

The number of Hox genes is variable within insects, with most variation owing to duplications of noncanonical Hox genes, especially the *zen* gene (the derived ortholog of the paralogy group 3 Hox gene) (Falciani et al. 1996). For example, the fruit fly *D. melanogaster* has three loci derived *zen* duplication: *zen*, *zen2,* and *bcd*, whereas *Tribolium castaneum* has two (*Tczen1*, *Tczen2*) (Brown et al. 2002). Several Lepidoptera have five zen-derived genes (*zen*, *ShxA*, *ShxB*, *ShxC*, *ShxD*) (Ferguson et al. 2014), with *B. mori* having around 15 (Chai et al. 2008; Ferguson et al. 2014). In contrast, during chordate evolution, tandem duplication of canonical Hox genes gave rise to 15 Hox genes in amphioxus and 14 in the common ancestor of vertebrates (Powers and Amemiya 2004; Holland et al. 2008). Genome duplications during vertebrate evolution increased the total number of Hox genes; for example, human and mouse have 39 Hox genes, the African butterfly fish *Pantodon buchholzi* has 45 Hox genes, the Atlantic eel *Anguilla anguilla* has 73 Hox genes, and the Atlantic salmon *Salmo salar* has 118 Hox genes and pseudogenes (Mungpakdee et al. 2008; Henkel et al. 2012; Martin and Holland 2014). Our analysis of Lepidoptera genomes has uncovered many cases of Hox gene duplication, including enormous arrays of Hox-derived loci. We find some moths have the highest number of Hox loci known to date.

We found two rare cases of single-gene tandem duplications in Lepidoptera, *ftz* in *S. lutea* (buff ermine moth) and *Dfd* in *Acronicta aceris* (sycamore moth), but otherwise, all variation in gene number was owing to gains and losses of zen-derived genes, including the Shx genes. Consistent with the study of Ferguson et al. (2014), it is true to a first approximation to say that most Lepidoptera have four Shx genes, plus *zen*, such that the full complement of Hox-derived genes is usually 14 (*lab*, *pb*, *zen*, *ShxA*, *ShxB*, *ShxC*, *ShxD*, *Dfd*, *Scr*, *Antp*, *ftz*, *Ubx*, *abd-A*, *Abd-B*). The minor exceptions we find to this rule include (1) a moth in the basal family Micropterygidae, which has multiple zen-derived genes, although these lack the distinctive amino acid signatures of Shx genes and are likely an independent duplication; (2) the six-spotted burnet moth, *Zygaena filipendulae*, with only two Shx genes, annotated as *ShxB* and *ShxC* (Supplemental Fig. S3); and (3) butterflies in family Lycaenidae and the genus *Melitaea*, which have each independently lost *ShxD* (although *M. cinxia* has four copies of Shx owing to a subsequent duplication of *ShxA*).

However, the biggest exceptions to the “four Shx” rule are the cases we find of independent, very extensive tandem duplication of Shx genes in several evolutionary lineages of moths. These expansions ranged from the seven copies in *S. costaestrigalis* (pinion-streaked snout moth) to an astonishing 165 loci found in *A. monoglypha* (dark arches moth). Other examples include 58 and 66 copies in *B. lacticolella* and *B. adustella*, respectively; 19 copies in *P. stratiotata* (Boyes et al. 2022a); 24 copies in *P. bucephala* (buff-tip moth) (Boyes et al. 2022b); 20 copies in *S. lutea* (buff ermine moth); and 34 copies in *N. fimbriata* (broad-bordered yellow underwing) (Holland et al. 2021). The particular Shx genes that underwent tandem duplication differed between species, with some showing duplication of single genes (e.g., *ShxD* in *P. stratiotata*, *N. pronuba*, and *I. aversata*; *ShxA* in *Z. pyrina*) and others having multiple copies of several of the four Shx genes (Supplemental Fig. S5). It is currently unclear whether these large gene arrays are adaptive, having been driven by selection, or whether they are neutral and a consequence of a genomic region prone to duplication. In other gene families, large changes in copy number have been found to be adaptive and related to certain environments or behaviors (Briscoe et al. 2013; Cheng et al. 2017; Rane et al. 2019; Chakraborty et al. 2021). The Shx genes are expressed in the serosa during development, an extraembryonic tissue implicated in innate immunity and desiccation resistance in insects (Panfilio 2008; Jacobs et al. 2013, 2014, 2022). It is therefore possible that Shx duplication is an adaptation associated with modifications to the egg, and indeed, many of the highly duplicated genes show increased rates of sequence evolution (Fig. 4A). One possibility is that specialization of multiple Shx genes permitted evolutionary refinement of serosal function, which may be important to the survival of lepidopteran eggs laid on exposed surfaces of vegetation or in other challenging niches (Holland et al. 2017). However, although some of the moth species with large Shx expansions do have unusual ecology (such as aquatic eggs in *P. stratiotata*), we have not found a common developmental pattern, environmental link, or egg laying behavior among all species with large tandem duplications of Shx genes.

The alternative hypothesis, that extensive tandem duplication of Shx genes is neutral, would demand an explanation for why the number of Shx genes is stable at four (or three) in most lepidopteran lineages, yet undergoes dramatic expansion in others. We do not find a pattern consistent with a widespread stochastic gain and loss: The pattern is one of either stability or expansion. We propose that such a pattern is indicative of an underlying mutational mechanism driving duplication in some species and not others. One possible mutational mechanism relates to transposable element content. In almost all species in which large tandem duplication occurs (14/19), we find significantly increased density of the LINE elements in the region containing the Shx genes relative to the rest of the Hox cluster (Fig. 5). Generally, transposon activity is highly regulated and reduced within the Hox cluster, owing to the importance of the order and structure of the genes for proper development (Fried et al. 2004). However, if LINE elements successfully invade the Hox gene cluster, they could potentially promote tandem gene duplication through nonhomologous pairing at meiosis. Thus, a neutral explanation could be that LINE elements invaded in some species and caused an increased rate of duplication mutations, without phenotypic effect.

The adaptive and the neutral hypotheses can be reconciled, because even if initial duplication is neutral, the new loci could be substrates for later adaptive evolution and the TEs themselves could alter gene regulation. By analogy, enrichment of TEs within the Hox gene clusters of *Anolis* lizards correlates with rates of speciation and affects the expression of Hox genes during development (Feiner 2016, 2019). It is interesting to note that invasion of TEs into *Anolis* lizard Hox clusters is not associated with gene duplication. This is possibly because all vertebrate Hox genes have anteroposterior expression domains that could be disrupted by tandem duplication; in Lepidoptera, the *zen* gene has lost ancestral regional expression and gained tissue-specific expression.

## Methods

### Data acquisition

The genome assemblies used in this analysis were produced by the Darwin Tree of Life Project (The Darwin Tree of Life Project Consortium 2022) and can be found under the European Nucleotide Archive (ENA; https://www.ebi.ac.uk/ena/browser/home) under accession number PRJEB40665 and on the Darwin Tree of Life (DToL) portal page (https://portal.darwintreeoflife.org). The genome for a non-ditrysian species was obtained from the recent sequencing of the Micropterigidae species *N. cornuta* (Li et al. 2021). Sequences for all homeodomains from three insects (*D. melanogaster*, *T. castaneum*, *Apis mellifera*) were downloaded from homeodb (Zhong et al. 2008; Zhong and Holland 2011; http://homeodb.zoo.ox.ac.uk). Sequences for the lepidopteran-specific special homeobox genes (Shx) were obtained from Ferguson et al. (2014). Summary of genomes used, their shortened names, family membership, GenBank accession IDs, and Project IDs are found in Supplemental Table S1.

### Homeobox gene identification

To identify homeobox genes in the assembled genomes, the homeodomain protein sequences were used as queries in a TBLASTN search against the lepidopteran genomes (*e*-value threshold of 1 × 10^−5^). Overlapping hits from the lepidopteran genomes were filtered to retain a single sequence per homeobox gene with the longest sequence match. The resulting sequences from the lepidopteran genomes were then subsequently used in a reciprocal BLASTX search against the homeodomain protein data set. For hits with significant percentage identity (>70%), the reciprocal BLAST search allowed for the initial identification of the given homeobox gene. A second round of sequence similarity searches was performed using MMseqs2 (Steinegger and Söding 2017) and 1 kb on either side of the homeobox genes annotated from the initial BLAST search. The scripts for each step are available at GitHub (https://github.com/PeterMulhair/HbxFinder). For divergent sequences, identification was performed using phylogenetic analysis (see Molecular Analysis of Homeobox Evolution). Visualization of the Hox gene clusters and gene tree used R 4.0.3 (R Core Team 2021) using gggenes (https://github.com/wilkox/gggenes) and ggtree (Yu et al. 2017), respectively. The newly identified homeodomain nucleotide sequences were then translated into an amino acid format using the sixpack package from EMBOSS (Madeira et al. 2019); amino acid sequences with the highest identity to known homeodomain sequences were retained.

### Homeobox gene expression

The expression of all homeobox genes identified in our data set of 123 species was assessed using whole-body RNA-seq data from a representative set of seven species (*Biston betularia*, *Limenitis camilla*, *Nymphalis urticae*, *Pararge aegeria*, *Pieris rapae*, *Vanessa atalanta*, *Vanessa cardui*). RNA-seq data were downloaded from the DToL portal page. Transcriptome assembly was performed for each species using Trinity v2.8.5 (Grabherr et al. 2011). Next, for each transcriptome assembly, the transcript abundance was calculated using kallisto v0.44 (Bray et al. 2016). Homeobox gene identification was then performed in each species using a reciprocal BLAST approach.

### Species tree inference

A species tree for the 123 lepidopteran species in our data set was generated using gene sets obtained from BUSCO v5.1.2 (Manni et al. 2021). First, genes were annotated using the Lepidoptera BUSCO gene sets. Next, the busco2phylo-nf pipeline (https://github.com/lstevens17/busco2phylo-nf) was used to extract FASTA files for each annotated gene, ensuring 100% species coverage in each one. Each gene was aligned using MAFFT v7.467 (Katoh et al. 2005), and gene trees were inferred using IQ-TREE v2.0 (Minh et al. 2020), using ModelFinder to find the model of best fit (Kalyaanamoorthy et al. 2017). Finally, a species tree was inferred using the supertree approach in ASTRAL v5.7.7 (Zhang et al. 2018).

### Molecular analysis of homeobox evolution

Phylogenetic reconstruction was performed using the homeodomain amino acid sequences Homeodomain sequences were aligned using MAFFT v7.467 (Katoh et al. 2005), and maximum likelihood trees were built using IQ-TREE v2.0 (Nguyen et al. 2015) and the LG + G model of sequence evolution. Tree visualization was performed using ggtree (Yu et al. 2017). To test for changes in rates of homeodomain sequence evolution of the Shx genes between the Lycaenidae species (which lost *ShxD*) and all other lepidopteran species with a normal set of Shx genes, we measure pairwise identity between species as a proxy for evolutionary rate. This analysis was performed using PhyKIT with the phykit pairwise_identity command (Steenwyk et al. 2021). To measure whether selection was relaxed or intensified in any of the three remaining Shx genes on any of the Lycaenidae branches, we used the RELAX model (Wertheim et al. 2015) implemented in HyPhy (Kosakovsky Pond et al. 2020).

### Hi-C data processing and TAD identification

Hi-C reads were mapped to the genomes using BWA 0.7.5a-r405 (Li 2013). HiCExplorer was then used to process the Hi-C data to form interaction maps, annotate the TADs and visualize the results (Ramírez et al. 2018).

### Repeat annotation and TE density analysis

TEs were annotated using both the RepeatModeler and RepeatMasker pipelines. For each genome tested, a de novo repeat library was generated from the genome assemblies using RepeatModeler2 (Flynn et al. 2020). This library was combined with the RepeatMasker Insecta library (Bao et al. 2015) and the SINE database (Vassetzky and Kramerov 2013) and was filtered for any protein-coding genes and repeat elements below 50 bases in length. Repeats were classified using RepeatMasker v4.1.0 (Smit et al. 2013–2015), and regions containing LINE, SINE, LTR, and DNA elements were extracted for subsequent analysis. Next, for each of the four broad TE classes, densities in 5-kb windows were calculated first for the regions containing the Shx genes and second for the full Hox gene cluster minus the Shx gene region and *lab*. Enrichment for TE density in the Shx gene region compared with the remaining Hox cluster was performed for each TE class using the Wilcoxon rank-sum test with Bonferroni correction in the SciPy Python package (Virtanen et al. 2020). TE density enrichment across the Lepidoptera phylogeny was visualized using the Toytree Python package (Eaton 2020). These analyses were not intended as exhaustive but to give insight into TE density within the Hox gene cluster.

## Data access

All data and code required to reproduce analyses and figures can be found in the Supplemental Materials and at GitHub (https:// github.com/PeterMulhair/Lepidoptera_homeobox) and Zenodo (https://zenodo.org/record/7274111).

## Supplementary Material

Supplemental Material

## References

[GR277118MULC1] Aase-Remedios ME, Ferrier DEK. 2021. Improved understanding of the role of gene and genome duplications in chordate evolution with new genome and transcriptome sequences. Front Ecol Evol 9: 703163. 10.3389/fevo.2021.703163

[GR277118MULC2] Acemel RD, Maeso I, Gómez-Skarmeta JL. 2017. Topologically associated domains: a successful scaffold for the evolution of gene regulation in animals. Wiley Interdiscip Rev Dev Biol 6: e265. 10.1002/wdev.265 28251841

[GR277118MULC3] Bao W, Kojima KK, Kohany O. 2015. Repbase Update, a database of repetitive elements in eukaryotic genomes. Mob DNA 6: 11. 10.1186/s13100-015-0041-9 26045719 PMC4455052

[GR277118MULC4] Boyes D, Chadd R, Mulhair P, University of Oxford and Wytham Woods Genome Acquisition Lab, Natural History Museum Genome Acquisition Lab, Darwin Tree of Life Barcoding collective, Wellcome Sanger Institute Tree of Life programme, Wellcome Sanger Institute Scientific Operations: DNA Pipelines collective, Tree of Life Core Informatics collective, Darwin Tree of Life Consortium. 2022a. The genome sequence of the ringed China-mark, *Parapoynx stratiotata* (Linnaeus, 1758). Wellcome Open Res 7: 121. 10.12688/wellcomeopenres.17808.1

[GR277118MULC5] Boyes D, Holland PWH, University of Oxford and Wytham Woods Genome Acquisition Lab, Darwin Tree of Life Barcoding collective, Wellcome Sanger Institute Tree of Life programme, Wellcome Sanger Institute Scientific Operations: DNA Pipelines collective, Tree of Life Core Informatics collective, Darwin Tree of Life Consortium. 2022b. The genome sequence of the buff-tip, *Phalera bucephala* (Linnaeus, 1758). Wellcome Open Res 7: 28. 10.12688/wellcomeopenres.17539.1

[GR277118MULC6] Boyes D, Holland PWH, University of Oxford and Wytham Woods Genome Acquisition Lab, Darwin Tree of Life Barcoding collective, Wellcome Sanger Institute Tree of Life programme, Wellcome Sanger Institute Scientific Operations: DNA Pipelines collective, Tree of Life Core Informatics collective, Darwin Tree of Life Consortium. 2022c. The genome sequence of the silver Y moth, *Autographa gamma* (Linnaeus, 1758). Wellcome Open Res 7: 100. 10.12688/wellcomeopenres.17758.1 PMC11128053 38799510

[GR277118MULC7] Bray NL, Pimentel H, Melsted P, Pachter L. 2016. Near-optimal probabilistic RNA-seq quantification. Nat Biotechnol 34: 525–527. 10.1038/nbt.3519 27043002

[GR277118MULC8] Briscoe AD, Macias-Muñoz A, Kozak KM, Walters JR, Yuan F, Jamie GA, Martin SH, Dasmahapatra KK, Ferguson LC, Mallet J, 2013. Female behaviour drives expression and evolution of gustatory receptors in butterflies. PLoS Genet 9: e1003620. 10.1371/journal.pgen.1003620 23950722 PMC3732137

[GR277118MULC9] Brown SJ, Fellers JP, Shippy TD, Richardson EA, Maxwell M, Stuart JJ, Denell RE. 2002. Sequence of the *Tribolium castaneum* homeotic complex: the region corresponding to the *Drosophila melanogaster* antennapedia complex. Genetics 160: 1067–1074. 10.1093/genetics/160.3.1067 11901122 PMC1462024

[GR277118MULC10] Butts T, Holland PWH, Ferrier DEK. 2008. The urbilaterian Super-Hox cluster. Trends Genet 24: 259–262. 10.1016/j.tig.2007.09.006 18472178

[GR277118MULC11] Chai C-L, Zhang Z, Huang F-F, Wang X-Y, Yu Q-Y, Liu B-B, Tian T, Xia Q-Y, Lu C, Xiang Z-H. 2008. A genomewide survey of homeobox genes and identification of novel structure of the Hox cluster in the silkworm, *Bombyx mori*. Insect Biochem Mol Biol 38: 1111–1120. 10.1016/j.ibmb.2008.06.008 19280701

[GR277118MULC12] Chakraborty M, Ramaiah A, Adolfi A, Halas P, Kaduskar B, Ngo LT, Jayaprasad S, Paul K, Whadgar S, Srinivasan S, 2021. Hidden genomic features of an invasive malaria vector, *Anopheles stephensi*, revealed by a chromosome-level genome assembly. BMC Biol 19: 28. 10.1186/s12915-021-00963-z 33568145 PMC7876825

[GR277118MULC13] Cheng T, Wu J, Wu Y, Chilukuri RV, Huang L, Yamamoto K, Feng L, Li W, Chen Z, Guo H, 2017. Genomic adaptation to polyphagy and insecticides in a major east Asian noctuid pest. Nat Ecol Evol 1: 1747–1756. 10.1038/s41559-017-0314-4 28963452

[GR277118MULC14] Chipman AD, Ferrier DEK, Brena C, Qu J, Hughes DST, Schröder R, Torres-Oliva M, Znassi N, Jiang H, Almeida FC, 2014. The first myriapod genome sequence reveals conservative arthropod gene content and genome organisation in the centipede *Strigamia maritima*. PLoS Biol 12: e1002005. 10.1371/journal.pbio.1002005 25423365 PMC4244043

[GR277118MULC15] Cook CE, Smith ML, Telford MJ, Bastianello A, Akam M. 2001. *Hox* genes and the phylogeny of the arthropods. Curr Biol 11: 759–763. 10.1016/S0960-9822(01)00222-6 11378385

[GR277118MULC16] The Darwin Tree of Life Project Consortium. 2022. Sequence locally, think globally: the Darwin tree of life project. Proc Natl Acad Sci 119: e2115642118. 10.1073/pnas.2115642118 35042805 PMC8797607

[GR277118MULC17] Duboule D. 2007. The rise and fall of Hox gene clusters. Development 134: 2549–2560. 10.1242/dev.001065 17553908

[GR277118MULC18] Duboule D, Morata G. 1994. Colinearity and functional hierarchy among genes of the homeotic complexes. Trends Genet 10: 358–364. 10.1016/0168-9525(94)90132-5 7985240

[GR277118MULC19] Duncan I. 1987. The bithorax complex. Annu Rev Genet 21: 285–319. 10.1146/annurev.ge.21.120187.001441 3327467

[GR277118MULC20] Eaton DAR. 2020. Toytree: a minimalist tree visualization and manipulation library for Python. Methods Ecol Evol 11: 187–191. 10.1111/2041-210X.13313

[GR277118MULC21] Eres IE, Gilad Y. 2021. A TAD skeptic: Is 3D genome topology conserved? Trends Genet 37: 216–223. 10.1016/j.tig.2020.10.009 33203573 PMC8120795

[GR277118MULC22] Falciani F, Hausdorf B, Schröder R, Akam M, Tautz D, Denell R, Brown S. 1996. Class 3 Hox genes in insects and the origin of zen. Proc Natl Acad Sci 93: 8479–8484. 10.1073/pnas.93.16.8479 8710895 PMC38697

[GR277118MULC23] Feiner N. 2016. Accumulation of transposable elements in *Hox* gene clusters during adaptive radiation of *Anolis* lizards. Proc Biol Sci 283: 20161555. 10.1098/rspb.2016.1555 27733546 PMC5069512

[GR277118MULC24] Feiner N. 2019. Evolutionary lability in *Hox* cluster structure and gene expression in *Anolis* lizards. Evol Lett 3: 474–484. 10.1002/evl3.131 31636940 PMC6791295

[GR277118MULC25] Ferguson L, Marlétaz F, Carter J-M, Taylor WR, Gibbs M, Breuker CJ, Holland PWH. 2014. Ancient expansion of the Hox cluster in Lepidoptera generated four homeobox genes implicated in extra-embryonic tissue formation. PLoS Genet 10: e1004698. 10.1371/journal.pgen.1004698 25340822 PMC4207634

[GR277118MULC26] Ferrier DEK. 2016. Evolution of homeobox gene clusters in animals: the giga-cluster and primary vs. secondary clustering. Front Ecol Evol 4: 36. 10.3389/fevo.2016.00036

[GR277118MULC27] Ferrier DE, Akam M. 1996. Organization of the Hox gene cluster in the grasshopper, *Schistocerca gregaria*. Proc Natl Acad Sci 93: 13024–13029. 10.1073/pnas.93.23.13024 8917538 PMC24040

[GR277118MULC28] Ferrier DEK, Holland PWH. 2002. *Ciona intestinalis* ParaHox genes: evolution of Hox/ParaHox cluster integrity, developmental mode, and temporal colinearity. Mol Phylogenet Evol 24: 412–417. 10.1016/S1055-7903(02)00204-X 12220984

[GR277118MULC29] Finnerty JR, Martindale MQ. 1998. The evolution of the Hox cluster: insights from outgroups. Curr Opin Genet Dev 8: 681–687. 10.1016/S0959-437X(98)80037-3 9914202

[GR277118MULC30] Flynn JM, Hubley R, Goubert C, Rosen J, Clark AG, Feschotte C, Smit AF. 2020. RepeatModeler2 for automated genomic discovery of transposable element families. Proc Natl Acad Sci 117: 9451–9457. 10.1073/pnas.1921046117 32300014 PMC7196820

[GR277118MULC31] Fried C, Prohaska SJ, Stadler PF. 2004. Exclusion of repetitive DNA elements from gnathostome *Hox* clusters. J Exp Zool B Mol Dev Evol 302B: 165–173. 10.1002/jez.b.20007 15054859

[GR277118MULC32] Garcia-Fernàndez J. 2005. The genesis and evolution of homeobox gene clusters. Nat Rev Genet 6: 881–892. 10.1038/nrg1723 16341069

[GR277118MULC33] Grabherr MG, Haas BJ, Yassour M, Levin JZ, Thompson DA, Amit I, Adiconis X, Fan L, Raychowdhury R, Zeng Q, 2011. Full-length transcriptome assembly from RNA-Seq data without a reference genome. Nat Biotechnol 29: 644–652. 10.1038/nbt.1883 21572440 PMC3571712

[GR277118MULC34] Grbić M, Van Leeuwen T, Clark RM, Rombauts S, Rouzé P, Grbić V, Osborne EJ, Dermauw W, Ngoc PCT, Ortego F, 2011. The genome of *Tetranychus urticae* reveals herbivorous pest adaptations. Nature 479: 487–492. 10.1038/nature10640 22113690 PMC4856440

[GR277118MULC35] Henkel CV, Burgerhout E, de Wijze DL, Dirks RP, Minegishi Y, Jansen HJ, Spaink HP, Dufour S, Weltzien F-A, Tsukamoto K, 2012. Primitive duplicate Hox clusters in the European eel's genome. PLoS One 7: e32231. 10.1371/journal.pone.0032231 22384188 PMC3286462

[GR277118MULC36] Hill J, Rastas P, Hornett EA, Neethiraj R, Clark N, Morehouse N, de la Paz Celorio-Mancera M, Cols JC, Dircksen H, Meslin C, 2019. Unprecedented reorganization of holocentric chromosomes provides insights into the enigma of lepidopteran chromosome evolution. Sci Adv 5: eaau3648. 10.1126/sciadv.aau3648 31206013 PMC6561736

[GR277118MULC37] Holland PWH. 2015. Did homeobox gene duplications contribute to the Cambrian explosion? Zoological Lett 1: 1. 10.1186/s40851-014-0004-x 26605046 PMC4604119

[GR277118MULC38] Holland LZ, Albalat R, Azumi K, Benito-Gutiérrez E, Blow MJ, Bronner-Fraser M, Brunet F, Butts T, Candiani S, Dishaw LJ, 2008. The amphioxus genome illuminates vertebrate origins and cephalochordate biology. Genome Res 18: 1100–1111. 10.1101/gr.073676.107 18562680 PMC2493399

[GR277118MULC39] Holland PWH, Marlétaz F, Maeso I, Dunwell TL, Paps J. 2017. New genes from old: asymmetric divergence of gene duplicates and the evolution of development. Philos Trans R Soc Lond B Biol Sci 372: 20150480. 10.1098/rstb.2015.0480 27994121 PMC5182412

[GR277118MULC40] Holland PWH, University of Oxford and Wytham Woods Genome Acquisition Lab, Darwin Tree of Life Barcoding collective, Wellcome Sanger Institute Tree of Life programme, Wellcome Sanger Institute Scientific Operations: DNA Pipelines collective, Tree of Life Core Informatics collective, Darwin Tree of Life Consortium. 2021. The genome sequence of the broad-bordered yellow underwing, *Noctua fimbriata* (Schreber, 1759). Wellcome Open Res 6: 345. 10.12688/wellcomeopenres.17490.1

[GR277118MULC41] Jacobs CGC, Rezende GL, Lamers GEM, van der Zee M. 2013. The extraembryonic serosa protects the insect egg against desiccation. Proc Biol Sci 280: 20131082. 10.1098/rspb.2013.1082 23782888 PMC3712428

[GR277118MULC42] Jacobs CGC, Spaink HP, van der Zee M. 2014. The extraembryonic serosa is a frontier epithelium providing the insect egg with a full-range innate immune response. eLife 3: e04111. 10.7554/eLife.04111 25487990 PMC4358341

[GR277118MULC43] Jacobs CGC, van der Hulst R, Chen Y-T, Williamson RP, Roth S, van der Zee M. 2022. Immune function of the serosa in hemimetabolous insect eggs. Philos Trans R Soc Lond B Biol Sci 377: 20210266. 10.1098/rstb.2021.0266 36252212 PMC9574632

[GR277118MULC44] Jagla K, Bellard M, Frasch M. 2001. A cluster of *Drosophila* homeobox genes involved in mesoderm differentiation programs. Bioessays 23: 125–133. 10.1002/1521-1878(200102)23:2<125::AID-BIES1019>3.0.CO;2-C 11169585

[GR277118MULC45] Janoušek V, Laukaitis CM, Yanchukov A, Karn RC. 2016. The role of retrotransposons in gene family expansions in the human and mouse genomes. Genome Biol Evol 8: 2632–2650. 10.1093/gbe/evw192 27503295 PMC5631067

[GR277118MULC46] Kalyaanamoorthy S, Minh BQ, Wong TKF, von Haeseler A, Jermiin LS. 2017. ModelFinder: fast model selection for accurate phylogenetic estimates. Nat Methods 14: 587–589. 10.1038/nmeth.4285 28481363 PMC5453245

[GR277118MULC47] Katoh K, Kuma K-I, Toh H, Miyata T. 2005. MAFFT version 5: improvement in accuracy of multiple sequence alignment. Nucleic Acids Res 33: 511–518. 10.1093/nar/gki198 15661851 PMC548345

[GR277118MULC48] Kawahara AY, Plotkin D, Espeland M, Meusemann K, Toussaint EFA, Donath A, Gimnich F, Frandsen PB, Zwick A, Dos Reis M, 2019. Phylogenomics reveals the evolutionary timing and pattern of butterflies and moths. Proc Natl Acad Sci 116: 22657–22663. 10.1073/pnas.1907847116 31636187 PMC6842621

[GR277118MULC49] Kosakovsky Pond SL, Poon AFY, Velazquez R, Weaver S, Hepler NL, Murrell B, Shank SD, Magalis BR, Bouvier D, Nekrutenko A, 2020. HyPhy 2.5: a customizable platform for evolutionary hypothesis testing using phylogenies. Mol Biol Evol 37: 295–299. 10.1093/molbev/msz197 31504749 PMC8204705

[GR277118MULC50] Krefting J, Andrade-Navarro MA, Ibn-Salem J. 2018. Evolutionary stability of topologically associating domains is associated with conserved gene regulation. BMC Biol 16: 87. 10.1186/s12915-018-0556-x 30086749 PMC6091198

[GR277118MULC51] Lawniczak MKN, Durbin R, Flicek P, Lindblad-Toh K, Wei X, Archibald JM, Baker WJ, Belov K, Blaxter ML, Marques Bonet T, 2022. Standards recommendations for the Earth BioGenome Project. Proc Natl Acad Sci 119: e2115639118. 10.1073/pnas.2115639118 35042802 PMC8795494

[GR277118MULC52] Leite DJ, Baudouin-Gonzalez L, Iwasaki-Yokozawa S, Lozano-Fernandez J, Turetzek N, Akiyama-Oda Y, Prpic N-M, Pisani D, Oda H, Sharma PP, 2018. Homeobox gene duplication and divergence in arachnids. Mol Biol Evol 35: 2240–2253. 10.1093/molbev/msy125 29924328 PMC6107062

[GR277118MULC53] Lemons D, McGinnis W. 2006. Genomic evolution of Hox gene clusters. Science 313: 1918–1922. 10.1126/science.1132040 17008523

[GR277118MULC54] Lewis EB. 1978. A gene complex controlling segmentation in *Drosophila*. Nature 276: 565–570. 10.1038/276565a0 103000

[GR277118MULC55] Li H. 2013. Aligning sequence reads, clone sequences and assembly contigs with BWA-MEM. arXiv:1303.3997 [q-bio.GN]. https://arxiv.org/abs/1303.3997.

[GR277118MULC56] Li X, Ellis E, Plotkin D, Imada Y, Yago M, Heckenhauer J, Cleland TP, Dikow RB, Dikow T, Storer CG, 2021. First annotated genome of a mandibulate moth, *Neomicropteryx cornuta*, generated using PacBio HiFi sequencing. Genome Biol Evol 13: evab229. 10.1093/gbe/evab229 34599325 PMC8557830

[GR277118MULC57] Liao Y, Zhang X, Chakraborty M, Emerson JJ. 2021. Topologically associating domains and their role in the evolution of genome structure and function in *Drosophila*. Genome Res 31: 397–410. 10.1101/gr.266130.120 33563719 PMC7919452

[GR277118MULC58] Luke GN, Castro LFC, McLay K, Bird C, Coulson A, Holland PWH. 2003. Dispersal of NK homeobox gene clusters in amphioxus and humans. Proc Natl Acad Sci 100: 5292–5295. 10.1073/pnas.0836141100 12704239 PMC154338

[GR277118MULC59] Madeira F, Park YM, Lee J, Buso N, Gur T, Madhusoodanan N, Basutkar P, Tivey ARN, Potter SC, Finn RD, 2019. The EMBL-EBI search and sequence analysis tools APIs in 2019. Nucleic Acids Res 47: W636–W641. 10.1093/nar/gkz268 30976793 PMC6602479

[GR277118MULC60] Manni M, Berkeley MR, Seppey M, Simão FA, Zdobnov EM. 2021. BUSCO update: novel and streamlined workflows along with broader and deeper phylogenetic coverage for scoring of eukaryotic, prokaryotic, and viral genomes. Mol Biol Evol 38: 4647–4654. 10.1093/molbev/msab199 34320186 PMC8476166

[GR277118MULC61] Martin KJ, Holland PWH. 2014. Enigmatic orthology relationships between *Hox* clusters of the African butterfly fish and other teleosts following ancient whole-genome duplication. Mol Biol Evol 31: 2592–2611. 10.1093/molbev/msu202 24974377 PMC4166920

[GR277118MULC62] Mazza ME, Pang K, Reitzel AM, Martindale MQ, Finnerty JR. 2010. A conserved cluster of three PRD-class homeobox genes (*homeobrain*, *rx* and *orthopedia*) in the Cnidaria and Protostomia. Evodevo 1: 3. 10.1186/2041-9139-1-3 20849646 PMC2938728

[GR277118MULC63] McGinnis W, Krumlauf R. 1992. Homeobox genes and axial patterning. Cell 68: 283–302. 10.1016/0092-8674(92)90471-N 1346368

[GR277118MULC64] Minh BQ, Schmidt HA, Chernomor O, Schrempf D, Woodhams MD, von Haeseler A, Lanfear R. 2020. IQ-TREE 2: new models and efficient methods for phylogenetic inference in the genomic era. Mol Biol Evol 37: 1530–1534. 10.1093/molbev/msaa015 32011700 PMC7182206

[GR277118MULC65] Mitter C, Davis DR, Cummings MP. 2017. Phylogeny and evolution of Lepidoptera. Annu Rev Entomol 62: 265–283. 10.1146/annurev-ento-031616-035125 27860521

[GR277118MULC67] Mungpakdee S, Seo H-C, Angotzi AR, Dong X, Akalin A, Chourrout D. 2008. Differential evolution of the 13 Atlantic salmon Hox clusters. Mol Biol Evol 25: 1333–1343. 10.1093/molbev/msn097 18424774

[GR277118MULC68] Negre B, Ruiz A. 2007. HOM-C evolution in *Drosophila*: Is there a need for *Hox* gene clustering? Trends Genet 23: 55–59. 10.1016/j.tig.2006.12.001 17188778

[GR277118MULC69] Negre B, Casillas S, Suzanne M, Sánchez-Herrero E, Akam M, Nefedov M, Barbadilla A, de Jong P, Ruiz A. 2005. Conservation of regulatory sequences and gene expression patterns in the disintegrating *Drosophila Hox* gene complex. Genome Res 15: 692–700. 10.1101/gr.3468605 15867430 PMC1088297

[GR277118MULC070] Nguyen L-T, Schmidt HA, von Haeseler A, Minh BQ. 2015. IQ-TREE: a fast and effective stochastic algorithm for estimating maximum-likelihood phylogenies. Mol Biol Evol 32: 268–274. 10.1093/molbev/msu300 25371430 PMC4271533

[GR277118MULC70] Nong W, Cao J, Li Y, Qu Z, Sun J, Swale T, Yip HY, Qian PY, Qiu J-W, Kwan HS, 2020. Jellyfish genomes reveal distinct homeobox gene clusters and conservation of small RNA processing. Nat Commun 11: 3051. 10.1038/s41467-020-16801-9 32561724 PMC7305137

[GR277118MULC71] Pace RM, Grbić M, Nagy LM. 2016. Composition and genomic organization of arthropod Hox clusters. Evodevo 7: 11. 10.1186/s13227-016-0048-4 27168931 PMC4862073

[GR277118MULC72] Panfilio KA. 2008. Extraembryonic development in insects and the acrobatics of blastokinesis. Dev Biol 313: 471–491. 10.1016/j.ydbio.2007.11.004 18082679

[GR277118MULC73] Powers TP, Amemiya CT. 2004. Evidence for a Hox14 paralog group in vertebrates. Curr Biol 14: R183–R184. 10.1016/j.cub.2004.02.015 15028231

[GR277118MULC74] Powers TP, Hogan J, Ke Z, Dymbrowski K, Wang X, Collins FH, Kaufman TC. 2000. Characterization of the Hox cluster from the mosquito *Anopheles gambiae* (Diptera: culicidae). Evol Dev 2: 311–325. 10.1046/j.1525-142x.2000.00072.x 11256376

[GR277118MULC75] Ramírez F, Bhardwaj V, Arrigoni L, Lam KC, Grüning BA, Villaveces J, Habermann B, Akhtar A, Manke T. 2018. High-resolution TADs reveal DNA sequences underlying genome organization in flies. Nat Commun 9: 189. 10.1038/s41467-017-02525-w 29335486 PMC5768762

[GR277118MULC76] Rane RV, Ghodke AB, Hoffmann AA, Edwards OR, Walsh TK, Oakeshott JG. 2019. Detoxifying enzyme complements and host use phenotypes in 160 insect species. Curr Opin Insect Sci 31: 131–138. 10.1016/j.cois.2018.12.008 31109666

[GR277118MULC77] Ranz JM, González PM, Su RN, Bedford SJ, Abreu-Goodger C, Markow T. 2022. Multiscale analysis of the randomization limits of the chromosomal gene organization between Lepidoptera and Diptera. Proc Biol Sci 289: 20212183. 10.1098/rspb.2021.2183 35042416 PMC8767184

[GR277118MULC78] R Core Team. 2021. R: a language and environment for statistical computing. R Foundation for Statistical Computing, Vienna. https://www.R-project.org/.

[GR277118MULC79] Schoenfelder S, Fraser P. 2019. Long-range enhancer–promoter contacts in gene expression control. Nat Rev Genet 20: 437–455. 10.1038/s41576-019-0128-0 31086298

[GR277118MULC80] Sexton T, Yaffe E, Kenigsberg E, Bantignies F, Leblanc B, Hoichman M, Parrinello H, Tanay A, Cavalli G. 2012. Three-dimensional folding and functional organization principles of the *Drosophila* genome. Cell 148: 458–472. 10.1016/j.cell.2012.01.010 22265598

[GR277118MULC81] Smit AFA, Hubley R, Green P. 2013–2015. RepeatMasker Open-4.0. http://www.repeatmasker.org.

[GR277118MULC82] Soshnikova N, Dewaele R, Janvier P, Krumlauf R, Duboule D. 2013. Duplications of hox gene clusters and the emergence of vertebrates. Dev Biol 378: 194–199. 10.1016/j.ydbio.2013.03.004 23501471

[GR277118MULC83] Startek M, Szafranski P, Gambin T, Campbell IM, Hixson P, Shaw CA, Stankiewicz P, Gambin A. 2015. Genome-wide analyses of LINE–LINE-mediated nonallelic homologous recombination. Nucleic Acids Res 43: 2188–2198. 10.1093/nar/gku1394 25613453 PMC4344489

[GR277118MULC84] Steenwyk JL, Buida TJ, Labella AL, Li Y, Shen X-X, Rokas A. 2021. PhyKIT: a broadly applicable UNIX shell toolkit for processing and analyzing phylogenomic data. Bioinformatics 37: 2325–2331. 10.1093/bioinformatics/btab096 33560364 PMC8388027

[GR277118MULC85] Steinegger M, Söding J. 2017. MMseqs2 enables sensitive protein sequence searching for the analysis of massive data sets. Nat Biotechnol 35: 1026–1028. 10.1038/nbt.3988 29035372

[GR277118MULC86] Szabo Q, Jost D, Chang J-M, Cattoni DI, Papadopoulos GL, Bonev B, Sexton T, Gurgo J, Jacquier C, Nollmann M, 2018. TADs are 3D structural units of higher-order chromosome organization in *Drosophila*. Sci Adv 4: eaar8082. 10.1126/sciadv.aar8082 29503869 PMC5829972

[GR277118MULC87] Szabo Q, Bantignies F, Cavalli G. 2019. Principles of genome folding into topologically associating domains. Sci Adv 5: eaaw1668. 10.1126/sciadv.aaw1668 30989119 PMC6457944

[GR277118MULC88] Thybert D, Roller M, Navarro FCP, Fiddes I, Streeter I, Feig C, Martin-Galvez D, Kolmogorov M, Janoušek V, Akanni W, 2018. Repeat associated mechanisms of genome evolution and function revealed by the *Mus caroli* and *Mus pahari* genomes. Genome Res 28: 448–459. 10.1101/gr.234096.117 29563166 PMC5880236

[GR277118MULC89] Ulianov SV, Khrameeva EE, Gavrilov AA, Flyamer IM, Kos P, Mikhaleva EA, Penin AA, Logacheva MD, Imakaev MV, Chertovich A, 2016. Active chromatin and transcription play a key role in chromosome partitioning into topologically associating domains. Genome Res 26: 70–84. 10.1101/gr.196006.115 26518482 PMC4691752

[GR277118MULC90] Vassetzky NS, Kramerov DA. 2013. SINEBase: a database and tool for SINE analysis. Nucleic Acids Res 41: D83–D89. 10.1093/nar/gks1263 23203982 PMC3531059

[GR277118MULC91] Virtanen P, Gommers R, Oliphant TE, Haberland M, Reddy T, Cournapeau D, Burovski E, Peterson P, Weckesser W, Bright J, 2020. SciPy 1.0: fundamental algorithms for scientific computing in Python. Nat Methods 17: 261–272. 10.1038/s41592-019-0686-2 32015543 PMC7056644

[GR277118MULC92] Walldorf U, Kiewe A, Wickert M, Ronshaugen M, McGinnis W. 2000. *Homeobrain*, a novel paired-like homeobox gene is expressed in the *Drosophila* brain. Mech Dev 96: 141–144. 10.1016/S0925-4773(00)00380-4 10940637

[GR277118MULC93] Wertheim JO, Murrell B, Smith MD, Kosakovsky Pond SL, Scheffler K. 2015. RELAX: detecting relaxed selection in a phylogenetic framework. Mol Biol Evol 32: 820–832. 10.1093/molbev/msu400 25540451 PMC4327161

[GR277118MULC94] Wiens JJ, Lapoint RT, Whiteman NK. 2015. Herbivory increases diversification across insect clades. Nat Commun 6: 8370. 10.1038/ncomms9370 26399434 PMC4598556

[GR277118MULC95] Yasukochi Y, Ashakumary LA, Wu C, Yoshido A, Nohata J, Mita K, Sahara K. 2004. Organization of the Hox gene cluster of the silkworm, *Bombyx mori*: a split of the Hox cluster in a non-*Drosophila* insect. Dev Genes Evol 214: 606–614. 10.1007/s00427-004-0441-1 15490231

[GR277118MULC96] Yu G, Smith DK, Zhu H, Guan Y, Lam TT-Y. 2017. ggtree: an R package for visualization and annotation of phylogenetic trees with their covariates and other associated data. Methods Ecol Evol 8: 28–36. 10.1111/2041-210X.12628

[GR277118MULC97] Zhang C, Rabiee M, Sayyari E, Mirarab S. 2018. ASTRAL-III: polynomial time species tree reconstruction from partially resolved gene trees. BMC Bioinformatics 19: 153. 10.1186/s12859-018-2129-y 29745866 PMC5998893

[GR277118MULC98] Zhong Y-F, Holland PWH. 2011. HomeoDB2: functional expansion of a comparative homeobox gene database for evolutionary developmental biology. Evol Dev 13: 567–568. 10.1111/j.1525-142X.2011.00513.x 23016940 PMC3399086

[GR277118MULC99] Zhong Y-F, Butts T, Holland PWH. 2008. HomeoDB: a database of homeobox gene diversity. Evol Dev 10: 516–518. 10.1111/j.1525-142X.2008.00266.x 18803769

