# Supplementary material for: Diversity, duplication, and genomic organization of homeobox genes in Lepidoptera
Source: Genome Res. 2023 Jan;33(1):32–44. doi: 10.1101/gr.277118.122 (PMC9977156; doi:10.1101/gr.277118.122)
Supplement: Supplemental Material [file supp_33_1_32__DC1.html]

Diversity, duplication, and genomic organization of homeobox genes in Lepidoptera — Diversity, duplication, and genomic organization of homeobox genes in Lepidoptera — Supplemental Material 

# Diversity, duplication, and genomic organization of homeobox genes in Lepidoptera

## Supplemental Material

- Supplemental\_Code\_1.zip
- Supplemental\_Data\_.zip
- Supplemental\_Materials.pdf
