# Supplementary material for: Diversity, duplication, and genomic organization of homeobox genes in Lepidoptera
Source: Genome Res. 2023 Jan;33(1):32–44. doi: 10.1101/gr.277118.122 (PMC9977156; doi:10.1101/gr.277118.122)
Supplement: Supplemental Material [file supp_gr.277118.122_Supplemental_Materials.pdf]

Supplementary data - Diversity, duplication and genomic organisation of homeobox genes in Lepidoptera

Peter O. Mulhair[1], Liam Crowley[1], Douglas H. Boyes[1,2], Amber Harper[1,3], Owen T. Lewis[1], Darwin Tree of Life Consortium[4], and Peter W.H. Holland[1,*]

[1]Department of Zoology, University of Oxford, 11a Mansfield Road, Oxford OX1 3SZ, UK
[2]UK Centre for Ecology Hydrology, Wallingford, OX10 8BB, UK
[3]Current address: Department of Biological and Medical Sciences, Faculty of Health and Life Sciences, Oxford Brookes University, Oxford, UK
[*]Corresponding author: Peter W.H. Holland, peter.holland@zoo.ox.ac.uk

Supplementary Figures

Supplementary Figure S1: Lepidoptera species tree.
Supplementary Figure S2: Homeobox gene expression in whole body RNAseq.
Supplementary Figure S3: Structure of Hox gene cluster across Lepidoptera.
Supplementary Figure S4: TAD structures surrounding homeobox gene clusters.
Supplementary Figure S5: Large tandem duplications of *zen*/Shx genes.
Supplementary Figure S6: Genome size and TE content across Lepidoptera species.
Supplementary Figure S7: Role of LINE expansion in driving Shx tandem duplication in the Leopard moth *Zeuzera pyrina*.
Supplementary Figure S8: Structure and orientation of *Hbn*, *Rx*, *Otp* gene cluster across Lepidoptera.
Supplementary Figure S9: Structure of NK gene cluster within Lepidoptera.

Supplementary Tables

Supplementary Table S1: Species sampled and genome source information.
Supplementary Table S2: Hi-C source data used for annotation of TADs.

Supplementary Figures

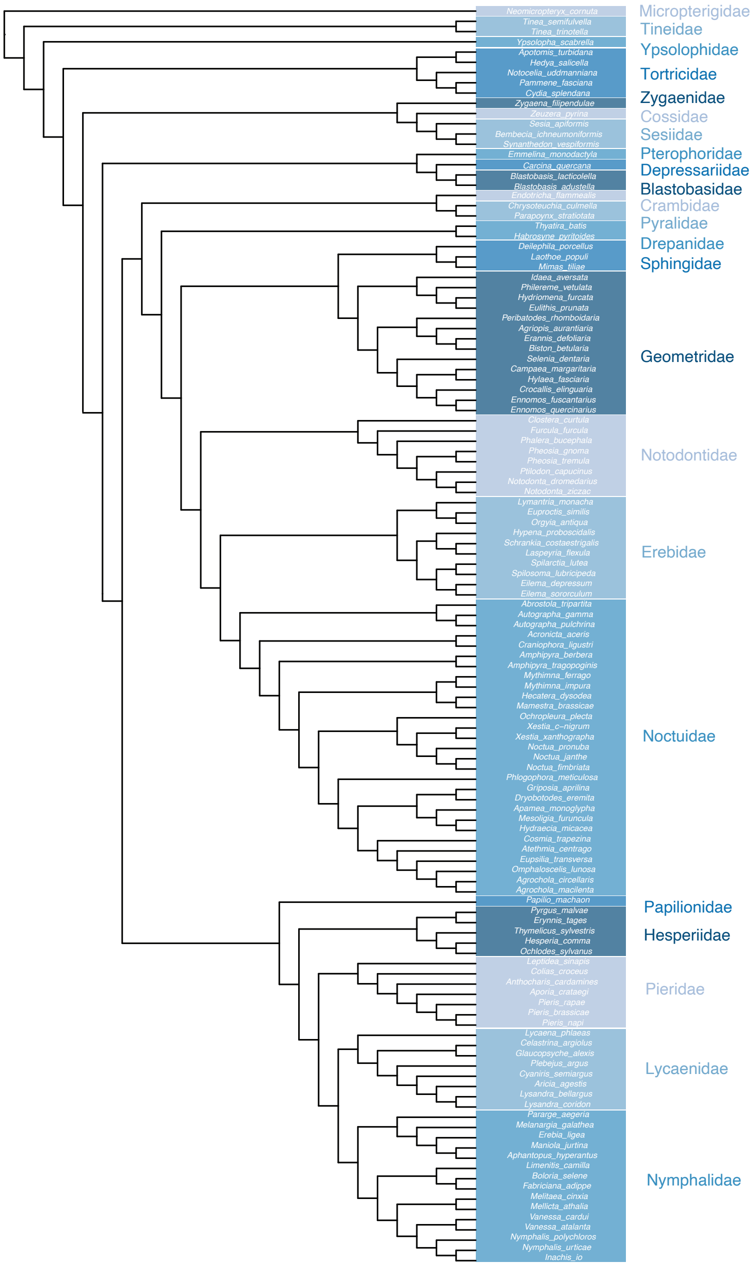

Supplemental Figure S1: Lepidoptera species tree. Species tree for representative Lepidoptera species inferred from BUSCO gene set using ASTRAL supertree approach. Species names in white are labelled by coloured boxes representing Lepidoptera families, with corresponding family names to the right. The family colours correspond to those in the species tree in Figure 1A in the main text.

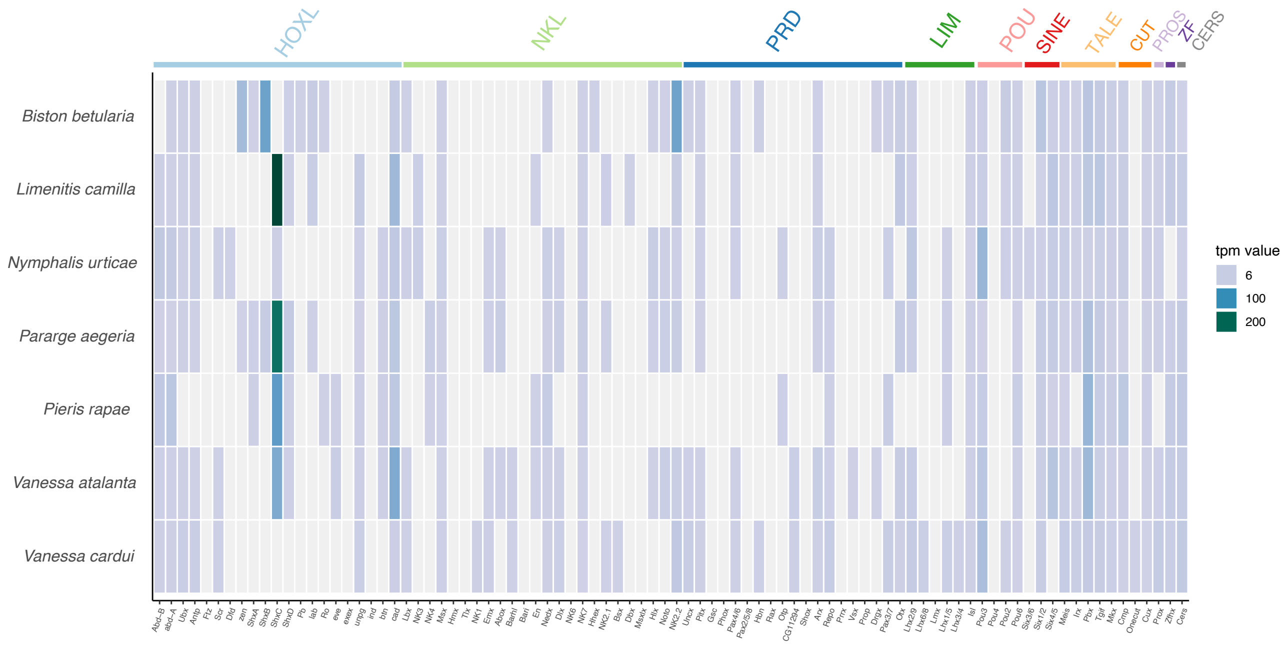

Supplemental Figure S2: Homeobox gene expression in whole body RNAseq. Gene expression quantification for all homeobox genes present in seven lepidopteran species. Whole body RNAseq data was taken from female individuals for each species shown. Each column represents a given homeobox gene, with the corresponding homeobox class to which it belongs shown at the top. Coloured cells represent level of gene expression in transcripts per million (tpm). Grey cells represent genes with no found expression.

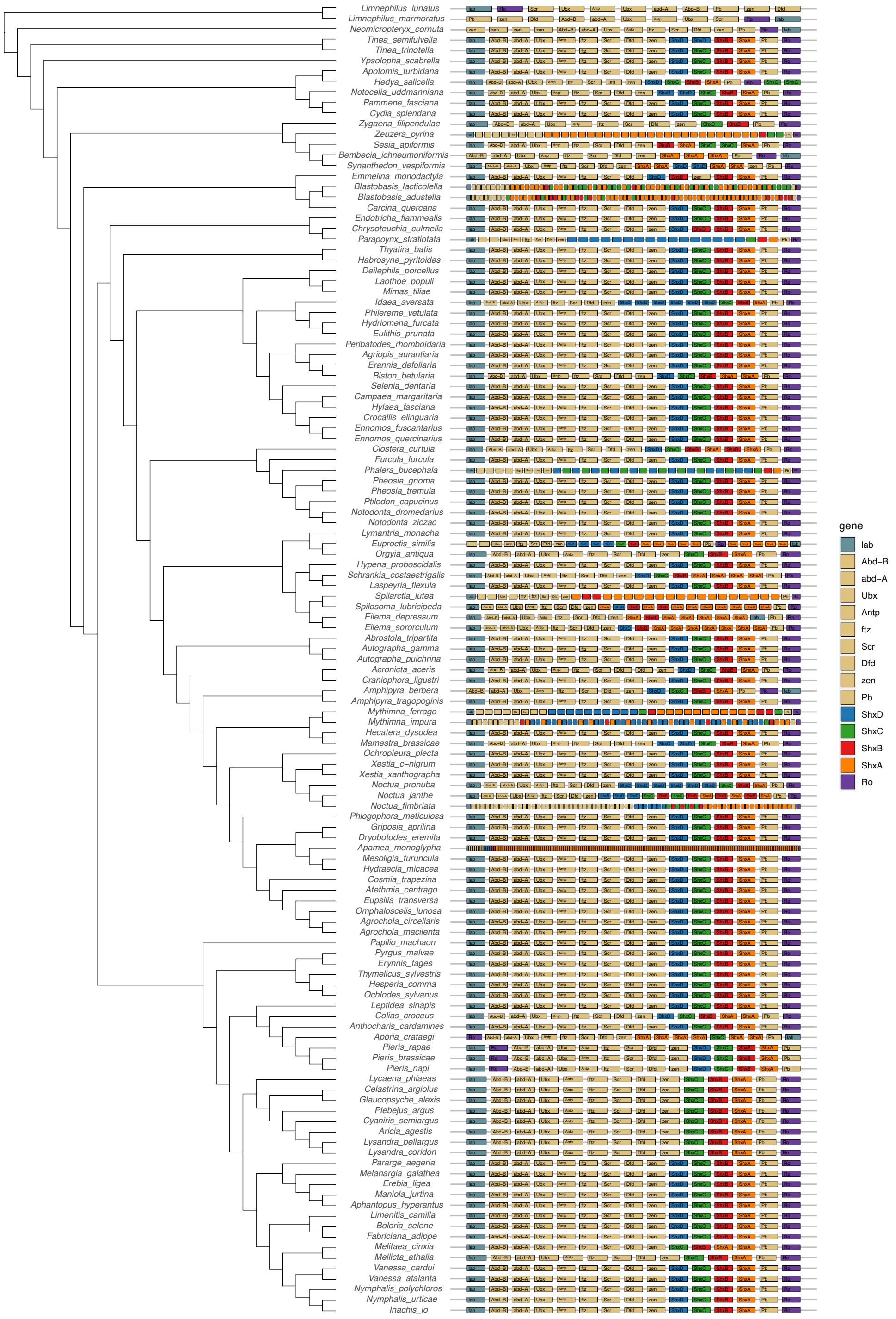

Supplemental Figure S3: Structure of Hox gene cluster across Lepidoptera. Left; Species tree topology of 123 Lepidoptera species along with 2 Trichoptera outgroup species. **Right;** Schematic of the Hox gene cluster in each species, with each box representing a homeobox gene (the name of each gene is present in the box where possible). The *labial* gene is coloured in grey-blue to indicate its dissociation from the Hox cluster. The Shx genes are coloured the same as is found in Figure 2 and 3 in the main text. The *rough* gene is coloured purple which indicates that it is a non-HOXL class gene that is associated with the Hox cluster in Lepidoptera.

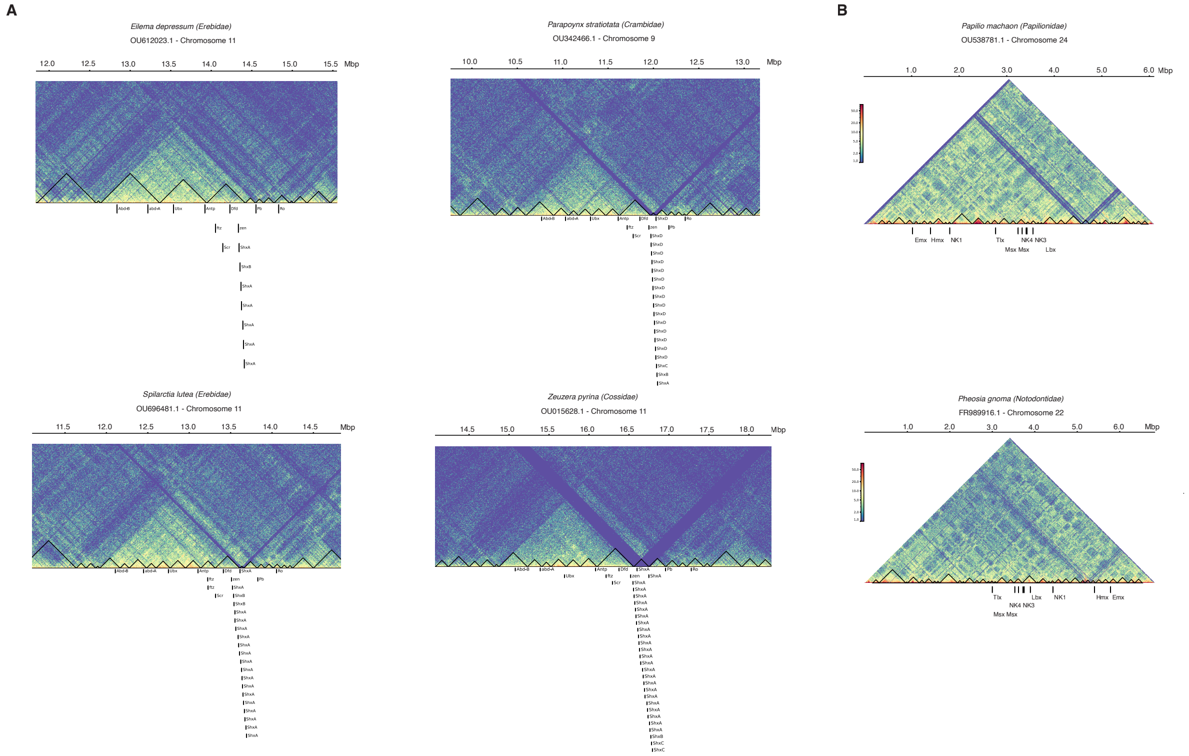

Supplemental Figure S4: TAD structures surrounding homeobox gene clusters. (A) Topological associated domains surrounding the Hox gene cluster for four species which have large number of Shx gene duplications. **(B)** TAD structures shown across the whole chromosome which contains the NK gene cluster for two species.

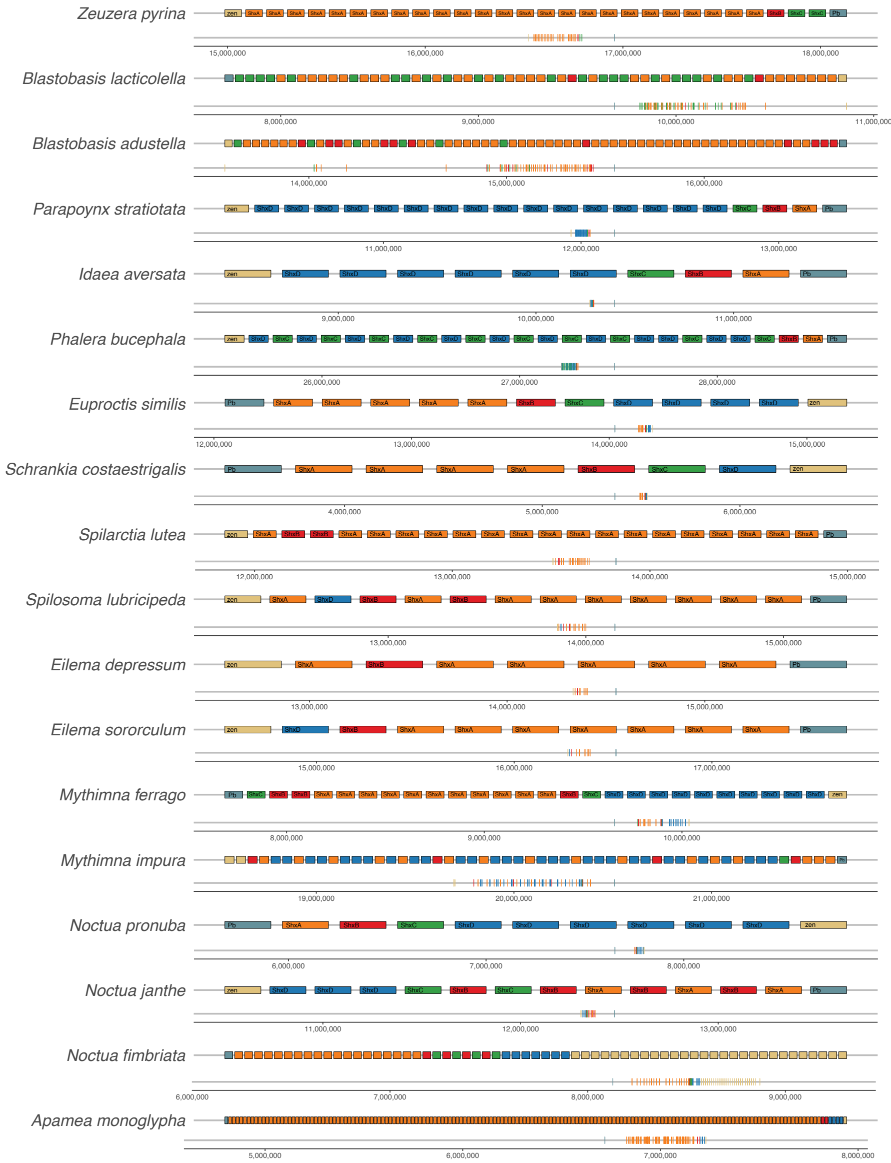

Supplemental Figure S5: Large tandem duplications of *zen*/Shx genes. Representation of 18 species which have large tandem duplications of the *zen*/Shx genes within the Hox cluster. Species names on the left are correlated with a schematic of the *zen*/Shx gene content (above) and the actual genomic locations of the genes shown (below).

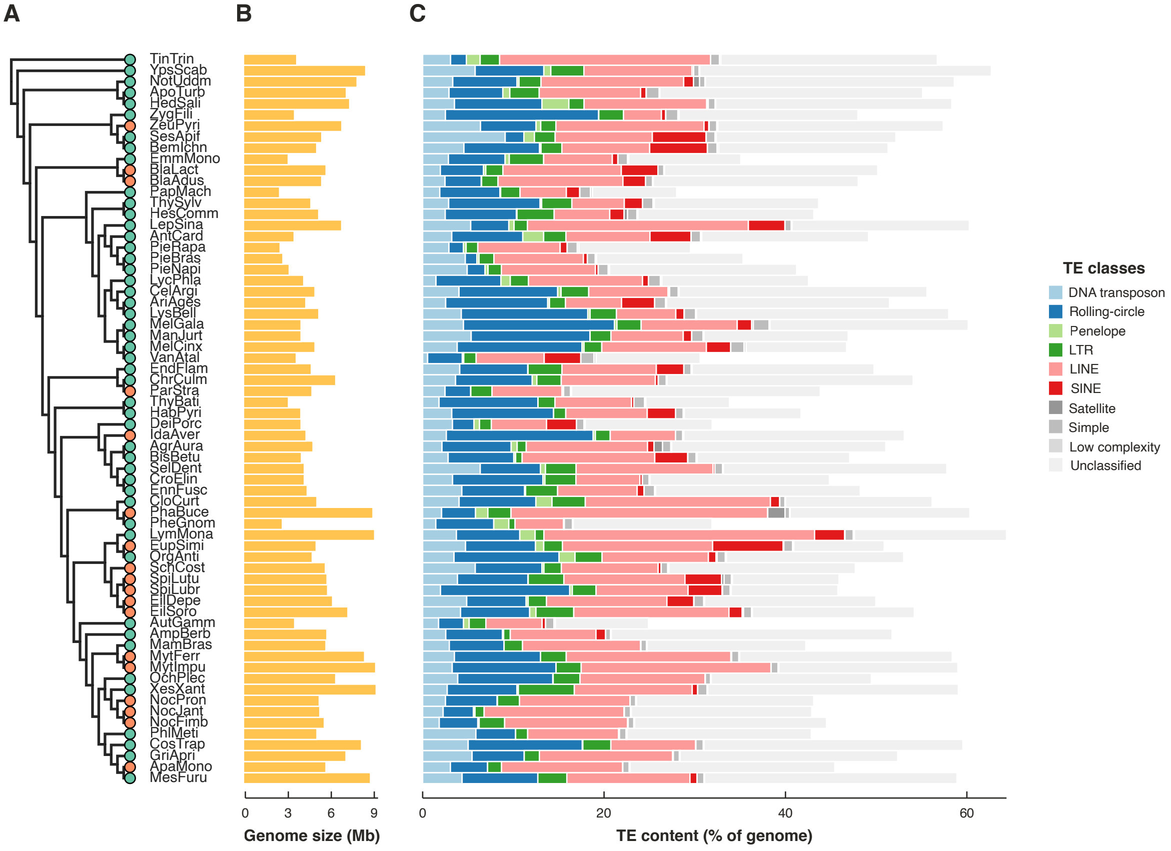

Supplemental Figure S6: Genome size and TE content across Lepidoptera species. (A) Species tree of the 66 lepidopteran species for which TE content was annotated. Dots at the tips of the tree represent species with a normal Shx gene count (green) and an expanded Shx gene count (orange). **(B)** Genome size for each species sampled, measured in megabases (Mb). **(C)** TE content as proportion of the genome, broken down by TE superfamily. Colours represent the different TE superfamilies (see figure legend).

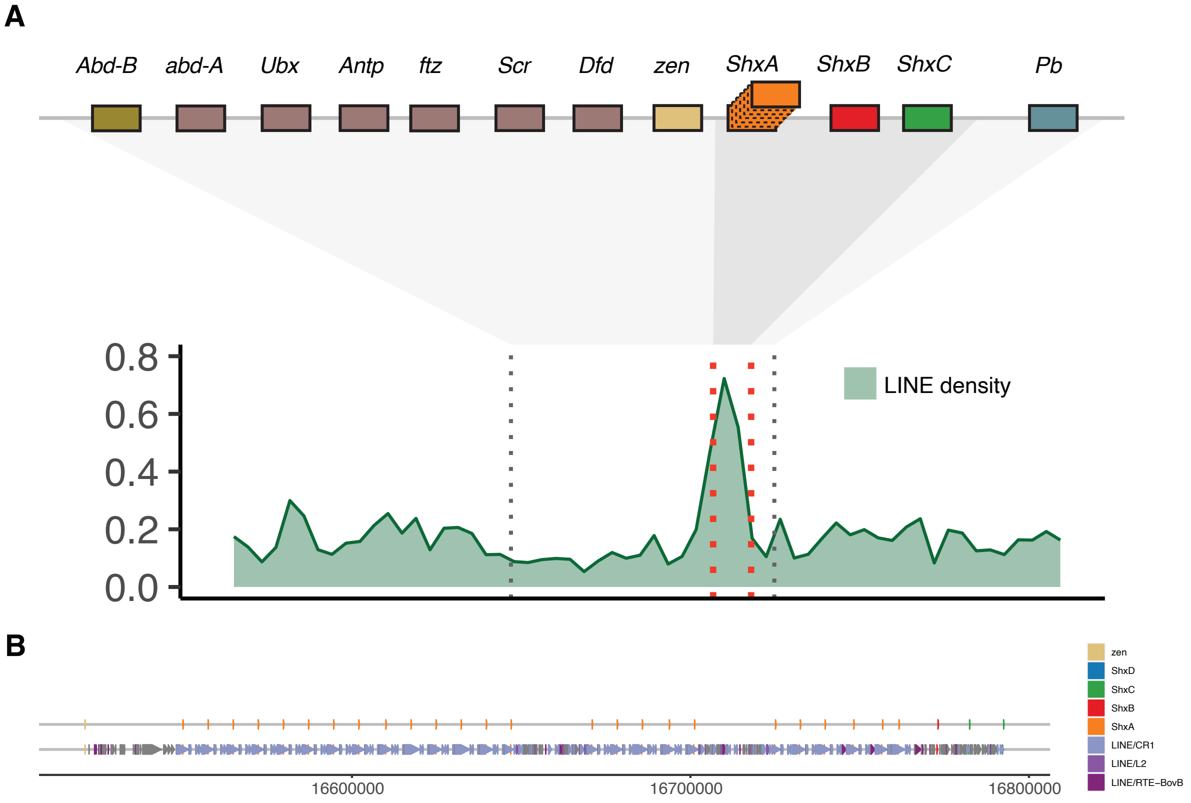

Supplemental Figure S7: Role of LINE expansion in driving Shx tandem duplication in the Leopard moth *Zeuzera pyrina*. (A) Above shows the order and content of the Hox gene cluster of this species, which has 25 copies of *ShxA*. Below shows the LINE density in 5kb windows across the Hox cluster. Shaded regions correlate to the Hox cluster shown above, with the full Hox cluster bordered by broken black lines and the Shx cluster bordered by red broken lines. **(B)** The *zen*/Shx cluster in its genomic location is shown above. Below shows the same region with LINE elements annotated, with orientation show by arrows. Genes and LINE elements are coloured according to the legend.

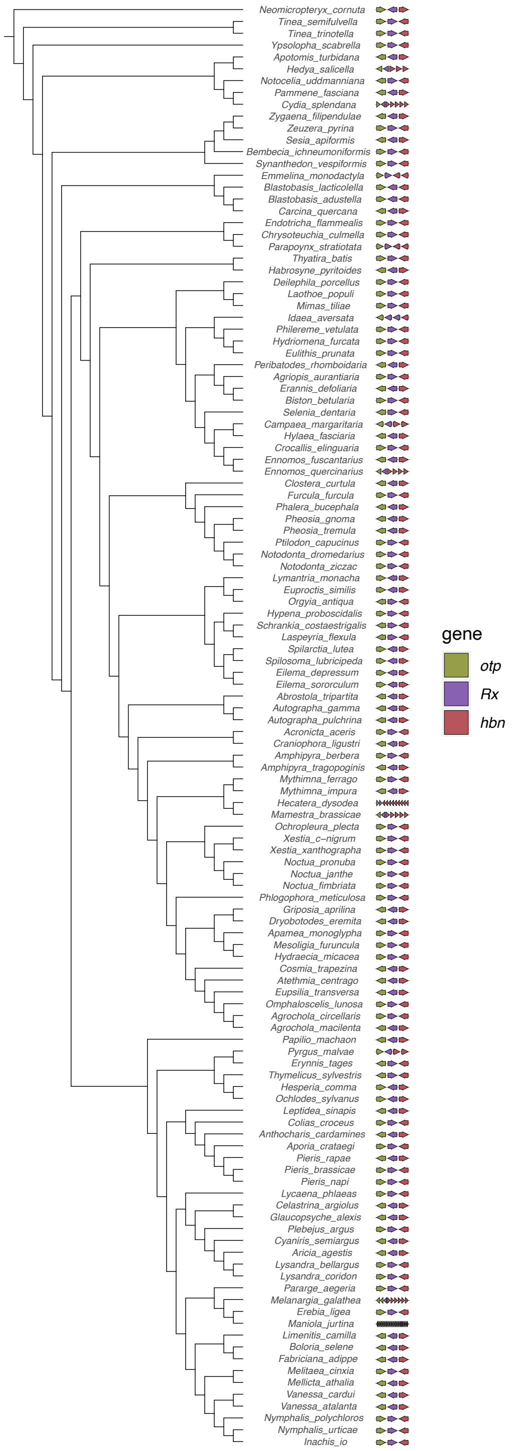

Supplemental Figure S8: Structure and orientation of *hbn*, *Rx*, *otp* gene cluster across Lepidoptera. **Left;** Species tree of 123 Lepidoptera species. **Right;** Schematic of the *hbn-Rx-otp* gene cluster in each species, with each box representing one of the three homeobox genes from the PRD class. Each of the three genes is coloured according to the legend. Gene orientation is signified by the direction of the arrow.

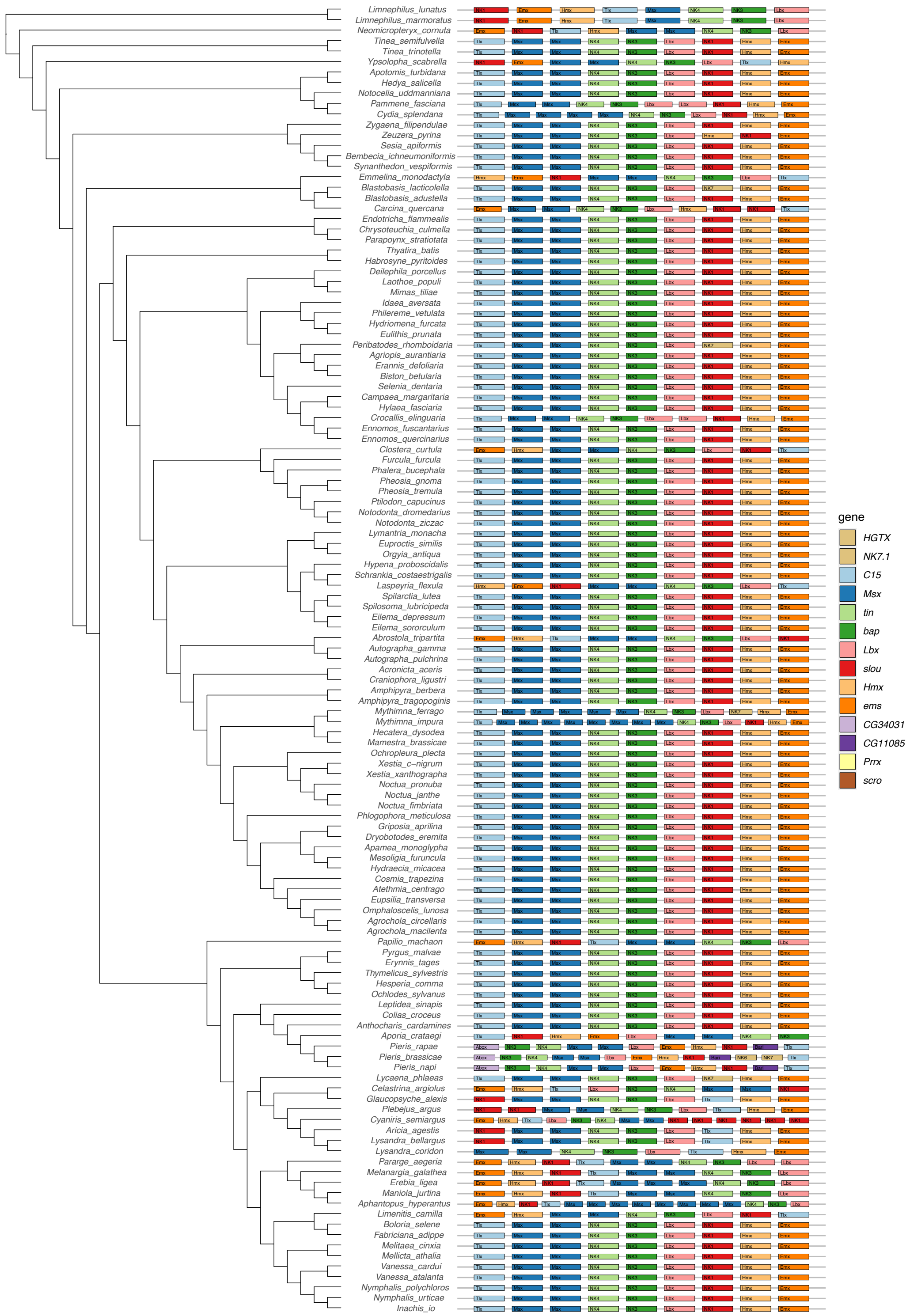

Supplemental Figure S9: Structure of NK gene cluster within Lepidoptera. Left; Species tree topology of 123 Lepidoptera species along with 2 Trichoptera outgroup species. **Right;** Schematic of the NK gene cluster in each species, with each box representing a homeobox gene (the name of each gene is present in the box where possible). Each gene in the NK cluster is coloured differently, with the colour and corresponding gene given in the figure legend. The gene colours are consistent with the colours found in Figure 6 and 7 in the main text.

Supplementary Tables

Table S1: Species sampled and genome source information.

Family	Species name	Short name	GenBank accession	Project ID
Erebidae	Euproctis similis	EupSimi	GCA_ 905147225	PRJEB40665
Erebidae	Hypena proboscidalis	HypProb	GCA_ 905147285	PRJEB40665
Erebidae	Spilosoma lubricipeda	SpiLubr	GCA_ 905220595	PRJEB40665
Erebidae	Eilema sororcula	EilSoro	GCA_ 914829495	PRJEB40665
Erebidae	Eilema depressum	EilDepe	GCA_ 914767945	PRJEB40665
Erebidae	Laspeyria flexula	LasFlex	GCA_ 905147015	PRJEB40665
Erebidae	Schrankia costaestrigalis	SchCost	GCA_ 905475405	PRJEB40665
Erebidae	Spilarctia lutea	SpiLutu	GCA_ 916048165	PRJEB40665
Erebidae	Lymantria monacha	LymMona	GCA_ 905163515	PRJEB40665
Erebidae	Orgyia antiqua	OrgAnti	GCA_ 916999025	PRJEB40665
Nymphalidae	Aphantopus hyperantus	AphHyp	GCA_ 902806685	PRJEB40665
Nymphalidae	Inachis io	AglIoxx	GCA_ 905147045	PRJEB40665
Nymphalidae	Nymphalis urticae	AglUrti	GCA_ 905147175	PRJEB40665
Nymphalidae	Maniola jurtina	ManJurt	GCA_ 905333055	PRJEB40665
Nymphalidae	Mellicta athalia	MelAtha	GCA_ 905220545	PRJEB40665
Nymphalidae	Melitaea cinxia	MelCinx	GCA_ 905220565	PRJEB40665
Nymphalidae	Nymphalis polychloros	NymPoly	GCA_ 905220585	PRJEB40665
Nymphalidae	Pararge aegeria	ParAegt	GCA_ 905163445	PRJEB40665
Nymphalidae	Vanessa atalanta	VanAtal	GCA_ 905147765	PRJEB40665
Nymphalidae	Vanessa cardui	VanCard	GCA_ 905220365	PRJEB40665
Nymphalidae	Boloria selene	BolSele	GCA_ 905231865	PRJEB40665
Nymphalidae	Erebia ligea	EreLige	GCA_ 917051295	PRJEB40665
Nymphalidae	Fabriciana adippe	FabAdip	GCA_ 905404265	PRJEB40665
Nymphalidae	Limenitis camilla	LimCami	GCA_ 905147385	PRJEB40665
Nymphalidae	Melanargia galathea	MelGala	GCA_ 920104075	PRJEB40665
Sphingidae	Hemaris fuciformis	HemFuc	GCA_ 907164795	PRJEB40665
Sphingidae	Laothoe populi	LaoPopu	GCA_ 905220505	PRJEB40665
Sphingidae	Mimas tiliae	MimTili	GCA_ 905332985	PRJEB40665
Sphingidae	Deilephila porcellus	DeiPorc	GCA_ 905220455	PRJEB40665
Noctuidae	Abrostola tripartita	AbrTrip	GCA_ 905340225	PRJEB40665
Noctuidae	Acronicta aceris	AcrAcer	GCA_ 910591435	PRJEB40665
Noctuidae	Amphipyra berbera	AmpBerb	GCA_ 910594945	PRJEB40665
Noctuidae	Noctua fimbriata	NocFimb	GCA_ 905163415	PRJEB40665
Noctuidae	Xestia xanthographa	XesXant	GCA_ 905147715	PRJEB40665
Noctuidae	Agrochola circellaris	AgrCirc	GCA_ 914767755	PRJEB40665
Noctuidae	Agrochola macilenta	AgrMaci	GCA_ 916701695	PRJEB40665
Noctuidae	Amphipyra tragopoginis	AmpTrag	GCA_ 905220435	PRJEB40665
Noctuidae	Apamea monoglypha	ApaMono	GCA_ 911387795	PRJEB40665
Noctuidae	Atethmia centrago	AteCent	GCA_ 905333075	PRJEB40665
Noctuidae	Autographa gamma	AutGamm	GCA_ 905146925	PRJEB40665
Noctuidae	Autographa pulchrina	AutPulc	GCA_ 905475315	PRJEB40665
Noctuidae	Cosmia trapezina	CosTrap	GCA_ 905163495	PRJEB40665

Noctuidae	Craniophora ligustri	CraLigu	GCA_ 905163465	PRJEB40665
Noctuidae	Dryobotodes eremita	DryErem	GCA_ 917490735	PRJEB40665
Noctuidae	Eupsilia transversa	EupTran	GCA_ 914767815	PRJEB40665
Noctuidae	Griposia aprilina	GriApri	GCA_ 916610205	PRJEB40665
Noctuidae	Hecatera dysodea	HecDyso	GCA_ 905332915	PRJEB40665
Noctuidae	Hydraecia micacea	HydMica	GCA_ 914767645	PRJEB40665
Noctuidae	Mamestra brassicae	MamBras	GCA_ 905163435	PRJEB40665
Noctuidae	Mesoligia furuncula	MesFuru	GCA_ 916614155	PRJEB40665
Noctuidae	Mythimna ferrago	MytFerr	GCA_ 910589285	PRJEB40665
Noctuidae	Mythimna impura	MytImpu	GCA_ 905147345	PRJEB40665
Noctuidae	Noctua janthe	NocJant	GCA_ 910589295	PRJEB40665
Noctuidae	Noctua pronuba	NocPron	GCA_ 905220335	PRJEB40665
Noctuidae	Ochropleura plecta	OchPlec	GCA_ 905475445	PRJEB40665
Noctuidae	Omphaloscelis lunosa	OmpLuno	GCA_ 916610215	PRJEB40665
Noctuidae	Phlogophora meticulosa	PhlMeti	GCA_ 905147745	PRJEB40665
Noctuidae	Xestia c-nigrum	XesCnig	GCA_ 916618015	PRJEB40665
Lycaenidae	Celastrina argiolus	CelArgi	GCA_ 905187575	PRJEB40665
Lycaenidae	Glaucopsyche alexis	GlaAlex	GCA_ 905404095	PRJEB40665
Lycaenidae	Lycaena phlaeas	LycPhla	GCA_ 905333005	PRJEB40665
Lycaenidae	Aricia agestis	AriAges	GCA_ 905147365	PRJEB40665
Lycaenidae	Cyaniris semiargus	CyaSemi	GCA_ 905187585	PRJEB40665
Lycaenidae	Lysandra bellargus	LysBell	GCA_ 905333045	PRJEB40665
Lycaenidae	Lysandra coridon	LysCori	GCA_ 905220515	PRJEB40665
Lycaenidae	Plebejus argus	PleArgu	GCA_ 905404155	PRJEB40665
Pieridae	Colias croceus	ColCroc	GCA_ 905220415	PRJEB40665
Pieridae	Pieris brassicae	PieBrab	GCA_ 905147105	PRJEB40665
Pieridae	Pieris napi	PieNapi	GCA_ 905231885	PRJEB40665
Pieridae	Pieris rapae	PieRapa	GCA_ 905147795	PRJEB40665
Pieridae	Anthocharis cardamines	AntCard	GCA_ 905404175	PRJEB40665
Pieridae	Aporia crataegi	ApoCrat	GCA_ 912999735	PRJEB40665
Pieridae	Leptidea sinapis	LepSina	GCA_ 905404315	PRJEB40665
Papilionidae	Papilio machaon	PapMach	GCA_ 912999745	PRJEB40665
Notodontidae	Notodonta dromedarius	NotDrom	GCA_ 905147325	PRJEB40665
Notodontidae	Phalera bucephala	PhaBuce	GCA_ 905147815	PRJEB40665
Notodontidae	Pheosia tremula	PheTrem	GCA_ 905333125	PRJEB40665
Notodontidae	Clostera curtula	CloCurt	GCA_ 905475355	PRJEB40665
Notodontidae	Furcula furcula	FurFurc	GCA_ 911728495	PRJEB40665
Notodontidae	Notodonta ziczac	NotZicz	GCA_ 918843915	PRJEB40665
Notodontidae	Pheosia gnoma	PheGnom	GCA_ 905404115	PRJEB40665
Notodontidae	Ptilodon capucinus	PtiCapc	GCA_ 914767695	PRJEB40665
Drepanidae	Thyatira batis	ThyBati	GCA_ 905147785	PRJEB40665
Drepanidae	Habrosyne pyritoides	HabPyri	GCA_ 907165245	PRJEB40665
Hesperiidae	Thymelicus sylvestris	ThySylv	GCA_ 911387775	PRJEB40665
Hesperiidae	Erynnis tages	EryTage	GCA_ 905147235	PRJEB40665
Hesperiidae	Hesperia comma	HesComm	GCA_ 905404135	PRJEB40665
Hesperiidae	Ochlodes sylvanus	OchSylv	GCA_ 905404295	PRJEB40665
Hesperiidae	Pyrgus malvae	PyrMalv	GCA_ 911387765	PRJEB40665

Tortricidae	Notocelia uddmanniana	NotUddm	GCA_ 905163555	PRJEB40665
Tortricidae	Apotomis turbidana	ApoTurb	GCA_ 905147355	PRJEB40665
Tortricidae	Cydia splendana	CydSple	GCA_ 910591565	PRJEB40665
Tortricidae	Hedya salicella	HedSali	GCA_ 905404275	PRJEB40665
Tortricidae	Pammene fasciana	PamFasc	GCA_ 911728535	PRJEB40665
Geometridae	Agriopis aurantiaria	AgrAura	GCA_ 914767915	PRJEB40665
Geometridae	Biston betularia	BisBetu	GCA_ 905404145	PRJEB40665
Geometridae	Campaea margaritaria	CamMarg	GCA_ 912999815	PRJEB40665
Geometridae	Crocallis elinguaria	CroElin	GCA_ 907269065	PRJEB40665
Geometridae	Ennomos fuscantarius	EnnFusc	GCA_ 905220475	PRJEB40665
Geometridae	Ennomos quercinarius	EnnQuei	GCA_ 910589525	PRJEB40665
Geometridae	Erannis defoliaria	EraDefo	GCA_ 905404285	PRJEB40665
Geometridae	Eulithis prunata	EulPrun	GCA_ 918843925	PRJEB40665
Geometridae	Hydriomena furcata	HydFurc	GCA_ 912999785	PRJEB40665
Geometridae	Hylaea fasciaria	HylFasc	GCA_ 905147375	PRJEB40665
Geometridae	Idaea aversata	IdaAver	GCA_ 907269075	PRJEB40665
Geometridae	Peribatodes rhomboidaria	PerRhom	GCA_ 911728515	PRJEB40665
Geometridae	Philereme vetulata	PhiVetu	GCA_ 918857605	PRJEB40665
Geometridae	Selenia dentaria	SelDent	GCA_ 917880725	PRJEB40665
Sesiidae	Bembecia ichneumoniformis	BemIchn	GCA_ 910589475	PRJEB40665
Sesiidae	Sesia apiformis	SesApif	GCA_ 914767545	PRJEB40665
Sesiidae	Synanthedon vespiformis	SynVesp	GCA_ 918317495	PRJEB40665
Blastobasidae	Blastobasis adustella	BlaAdus	GCA_ 907269095	PRJEB40665
Blastobasidae	Blastobasis lacticolella	BlaLact	GCA_ 905147135	PRJEB40665
Depressariidae	Carcina quercana	CarQuer	GCA_ 910589575	PRJEB40665
Crambidae	Chrysoteuchia culmella	ChrCulm	GCA_ 910589605	PRJEB40665
Crambidae	Parapoynx stratiotata	ParStra	GCA_ 910589355	PRJEB40665
Pterophoridae	Emmelina monodactyla	EmmMono	GCA_ 916618145	PRJEB40665
Pyralidae	Endotricha flammealis	EndFlam	GCA_ 905163395	PRJEB40665
Tineidae	Tinea semifulvella	TinSemi	GCA_ 910589645	PRJEB40665
Tineidae	Tinea trinotella	TinTrin	GCA_ 905220615	PRJEB40665
Ypsolophidae	Ypsolopha scabrella	YpsScab	GCA_ 910592155	PRJEB40665
Cossidae	Zeuzera pyrina	ZeuPyri	GCA_ 907165235	PRJEB40665
Zygaenidae	Zygaena filipendulae	ZygFili	GCA_ 907165275	PRJEB40665
Micropterigidae	Neomicropteryx cornuta	NeoCorn	GCA_ 020383195	PRJNA731916

Table S2: Hi-C source data used for annotation of TADs.

Species	SRA ID	No. of reads
Deilephila porcellus	ERR6054400	47,309,104
Biston betularia	ERR6054591	41,236,939
Pheosia gnoma	ERR6054689	46,595,383
Acronicta aceris	ERR6054960	35,160,601
Agrochola circellaris	ERR6688510	37,177,903
Papilio machaon	ERR6363331	44,525,262
Colias croceus	ERR6054398	41,268,982
Lysandra bellargus	ERR6054517	45,917,117
Boloria selene	ERR6054470	52,924,894
